# Programmable In Vivo Selection of Arbitrary DNA Sequences

**DOI:** 10.1371/journal.pone.0047795

**Published:** 2012-11-14

**Authors:** Tuval Ben Yehezkel, Tamir Biezuner, Gregory Linshiz, Yair Mazor, Ehud Shapiro

**Affiliations:** 1 Department of Computer Science and Applied Mathematics, Weizmann Institute of Science, Rehovot, Israel; 2 Department of Biological Chemistry Weizmann Institute of Science, Rehovot, Israel; Center for Genomic Regulation, Spain

## Abstract

The extraordinary fidelity, sensory and regulatory capacity of natural intracellular machinery is generally confined to their endogenous environment. Nevertheless, synthetic bio-molecular components have been engineered to interface with the cellular transcription, splicing and translation machinery in vivo by embedding functional features such as promoters, introns and ribosome binding sites, respectively, into their design. Tapping and directing the power of intracellular molecular processing towards synthetic bio-molecular inputs is potentially a powerful approach, albeit limited by our ability to streamline the interface of synthetic components with the intracellular machinery in vivo. Here we show how a library of synthetic DNA devices, each bearing an input DNA sequence and a logical selection module, can be designed to direct its own probing and processing by interfacing with the bacterial DNA mismatch repair (MMR) system *in vivo* and selecting for the most abundant variant, regardless of its function. The device provides proof of concept for programmable, function-independent DNA selection *in vivo* and provides a unique example of a logical-functional interface of an engineered synthetic component with a complex endogenous cellular system. Further research into the design, construction and operation of synthetic devices *in vivo* may lead to other functional devices that interface with other complex cellular processes for both research and applied purposes.

## Introduction

Although the cellular machinery is orders of magnitude more complex than any synthetic biological device produced so far [1]–[3], the processing power of the vast majority of endogenous bio-molecular machines has not been harnessed by synthetic devices. This is partly due to the fact that even simple synthetic molecular devices have proven difficult to operate inside cells due to constraints placed by the highly evolved and optimized cellular environment. Nevertheless, considerable progress has been made in recent years by us and others in developing devices with the capacity to sense biomolecular entities compute cellular states and link these operations to varied outputs [4]–[11].

Synthetic biology often assimilates existing knowledge gained through basic research into new functional synthetic components and/or systems [12], [13]. In this report we describe a utilization of the comprehensive understanding of bacterial mismatch repair molecular biology [14]–[17] for the design of a functional device.

**Figure 1:**
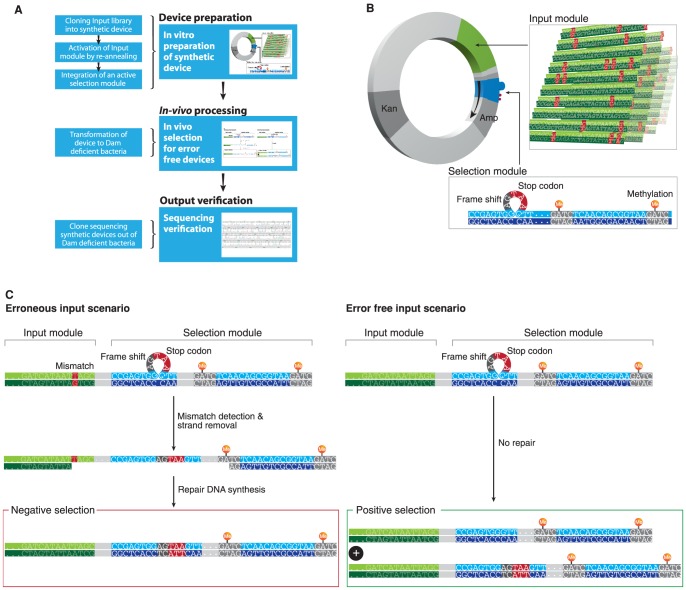
Overview of structure and operation principle of the synthetic device. A. The synthetic device is assembled *in vitro* using a 3-step process (top). It is then transformed to *E. coli* and processed *in vivo* by the MMR system according to the device's operating principles (middle). Finally, the output of the process is analyzed *in vitro* by purifying the devices out of bacteria and DNA sequencing them (bottom) **B.** Description of device components: the device library consists of (1) an input module containing many different variants of the same gene (green) and (2) a Selection module (blue) integrated within an Amp resistance gene (gray). The selection module contains a loop on its coding strand which frame-shifts (dark gray) and stops the translation (red stop codon) of the Amp gene. The device also bears a Kan resistance gene for noise reduction purposes. **C.** Schematic flow of device operation *in vivo*: if no mismatch is detected by the MMR (right, error free input scenario) no repair takes place, both strands are replicated and the heterozygous bacteria will live. Otherwise, if a mismatch is detected by the MMR (left, erroneous input scenario) repair synthesis spans the mismatch and hemi-methylated Dam site using the methylated, disrupted Amp strand as template and results in cell death.

We introduce an approach to engineer a synthetic DNA device which directs the probing and processing power of the endogenous MMR machinery towards the selection of a specific, but arbitrary DNA sequence according to rules embedded within the device's sequence and structure (Figure 1). More concretely, the device utilizes the mismatch recognition mechanisms of bacteria to identify that a mismatch exists on the device. Next, if a mismatch is identified, a methylation pattern embedded within the DNA sequence of the device directs the bacterial mismatch repair mechanism to eliminate portions of the device that reprogram it to kill its host cell. If a mismatch is not identified at the first step, the device is not reprogrammed and its host cell will not be killed. This engineered mechanism enables the identification and selection for any dominant DNA sequence within a large pool of mutated sequences.

The device is a circular dsDNA molecule (Figure 1b) with two major functional modules: (1) an input module that contains two supposedly complementary members of a DNA library, which are screened by the MMR system for the presence of a mismatch between them and (2) a Selection module that directs MMR-mediated processing and reprogramming of the device in case mismatches are detected in the input module.

**Figure 2:**
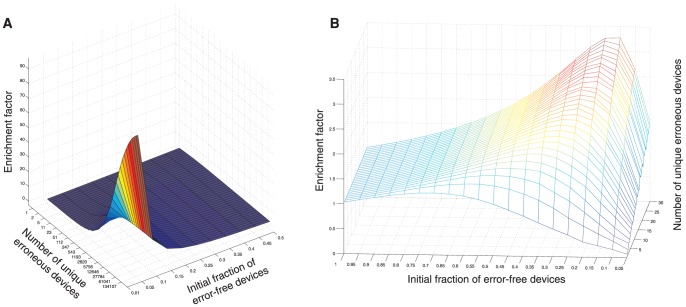
Simulation of the enrichment landscape following re-annealing mediated activation of the input module. **A.** The enrichment factor is computed as the ratio of correct (i.e. of the sequence we are enriching for) to total (correct+erroneous) homo-duplexes before re-annealing of the input module divided by the same ratio after its re-annealing. It is plotted as a function of the initial fraction of correct molecules and the number of unique erroneous molecules. **B.** magnified view of the specific zone in the enrichment space presented in A.

The Selection module is embedded within the coding region of an Ampicillin (Amp) resistance gene and contains two functional elements (Figure 1b): (1) a loop structure on one strand which disrupts the Amp gene with a frame shift and a stop codon (See Figure 1b) and (2) two adjacent synthetic hemi-methylated Dam (GATC) sites that protect the looped strand from MMR degradation. The synthetic hemi-methylations designate their strand as template for repair synthesis in case the input module contains a mismatch. MMR-mediated, hemi-methylation directed repair synthesis of the non-methylated strand spans the mismatch and hemi-methylated Dam sites [14], [15]. We utilized this natural feature of the MMR system to generate Ampicillin-sensitive bacteria in cases of erroneous input modules. Using this design principle we were able to use the Selection module as an apparatus that effectively pairs MMR-based detection of mismatches in the input module library to an overall positive selection for bacteria that carry error-free input modules (See Figure 1c).

More concretely, upon transformation of the device to bacteria the MutHLS machinery detects whether a mismatch is present in the input module. Positive (mutation) diagnosis induces (1) MMR scanning for the closest hemi-methylated Dam site within a 1 Kb range [14], [15], (2) selection of the disrupted methylated strands as template for repair synthesis [14], [15] and ultimately cell death (See Figure. 1c left panel). Negative diagnosis (no mismatch) does not activate the MMR system and results in replication of both strands, one of which encodes a functional Amp gene that rescues the cell (See Figure 1c right panel).

The current system design selects for the most abundant DNA sequence in a library of variants, as is the case for the fraction of error-free molecules in synthetic DNA construction [18]–[22] and other nucleic acid enrichment challenges in biology [23]–[25]. See **Description of experimental procedures** (Text S1) in supplementary material for a more thorough, chronological of experimental procedures and setup.

## Materials and Methods

### Chemical Oligonucleotide Synthesis

Oligonucleotides for all experiments were ordered from Sigma. Most of the oligonucleotides were standard desalted. Several oligonucleotides were labeled with 5′ fluorescent HEX or N-6 methyl-adenosine in Dam sites (GATC).

### Error-Prone PCR

Error-prone PCR was done using GeneMorph II Random Mutagenesis Kit (Stratagene) according to standard protocol except for Thermal Cycler program: Activation 95°C for 6 min, 15–19 cycles of: Denaturation 95°C for 30 sec, annealing at 55°C for 30 sec and extension 72°C for 1∶30 min.

### Electroporation

On ice, 1–2 µl of purified DNA, eluted in double deionized water (DDW) was mixed with 25 µl of electrocompetent bacterial cells. The mixture was transferred into an electroporation cuvette (BTX) followed by the employment of a 1.8 kV pulse using the Gene Pulser Xcell total system (Bio-Rad). After electroporation, 975 µl of SOC were added and a recovery step of 1 hour at 37°C was performed before inoculation into selective petri dishes.

### Sequencing

We used Sanger sequencing using the BigDye® Terminator v1.1 Cycle Sequencing Kit (ABI). We purified the sequencing reaction using the Performa® DTR Ultra 96-Well Plate Kit (EdgeBio) and analyzed the products using the ABI 3130 genetic Analyzer.

See Text S1 for additional materials and methods.

## Results

Mismatches between the various DNA molecules cloned into the input module were exposed by melting and re-annealing the input modules, which generated both hetero and homo-duplex input modules. The fractions of homo-duplex input modules are known since the concentrations of each input module variant in the mixture were controlled by us. We calculate that the small differences in sequence between input modules had a negligible effect on the Tm between the different variants (see sequences in Text S2) and hence the re-annealed population is the result of random re-annealing, from which the hetero-duplex fraction can be inferred. As our device selects against hetero-duplex input modules we simulated the fraction of post-annealing error-free homo-duplexes compared to their initial, pre-annealing fraction of the population as a function of two parameters: (1) the initial fraction of devices with the sequence being selected for and (2) the total number of unique erroneous variants. Our results show that, within specific constraints of these two parameters, mismatch-free devices with erroneous input modules are less frequent compared to mismatch-free devices with the correct input module (Figure 2, Text S3, Figure S1). The enrichment landscape shows that enrichment ratio is high when the initial fraction of devices with the sequence being selected for is low and the total number of unique erroneous variants is high (Figure 2), as long as the sequence being selected for is the dominant species.

**Figure 3:**
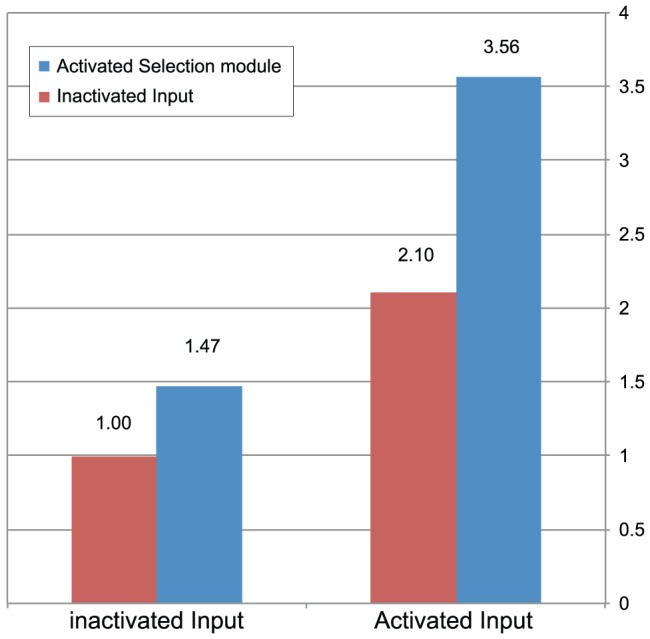
Summary of sequencing results and enrichment factors. The sequencing results of the input module from several hundred purified devices from Dam deficient bacteria are summarized and presented as the experimental enrichment factor of the device and several controls. Devices with activated and inactivated input modules (on the right and left, respectively) were tested with activated and inactivated Selection modules (blue and red, respectively). See text for explanations of activated and inactivated modules. They were then clone sequenced and their enrichment factor is presented on the Y axis. The results (from table 1) were normalized so that enrichment observed in experiments was compared to the results of the no-annealing, no-selection experiment.

This principle forms the basis for MMR mediated selection for devices with the correct input module sequence. Population constraints at which the enrichment factor for error-free homo-duplex input modules is significant are typical “needle in a haystack” problems in biology in which one seeks to identify/obtain a specific, scarce genetic element within a vastly larger population of background/noise genetic elements. These include, among others, the population of error-free DNA molecules in *de novo* DNA construction (i.e. when there are many more erroneous than error-free molecules) [18]–[22] and the population of specifically mutated DNA/RNA molecules within larger pools of un-mutated DNA/RNA *in vivo* or in culture [23]–[25]. These simulation results, as well as the basic operating principle of our device (Figure 1c), predict that re-annealing based activation of the input module should be critical for proper operation since it (1) exposes mismatches between input module library members and (2) enriches the correct DNA sequence fraction of the (post annealing) homo-duplex population. We confirmed these predictions experimentally by showing that efficient *in vivo* operation of the device is re-annealing dependent (See Figure 3 and Table 1).

**Table 1:** Comparative summary of experimental results from various devices.

	Reference device		Tested device		Enrichment		
	Input module	Selection module	Input module	Selection module	Deviation from expected error free fraction	P-value	Devices clone-sequenced
1	−	−	+	+	356%	6.10E-05	172
2	−	−	+	−	210%	6.20E-04	190
3	+	−	+	+	169%	2.40E-02	176
4	−	−	−	+	147%	7.40E-02	189

The table describes four comparisons (rows 1–4) between the enrichment factor resulting from devices with various combinations of activated and inactivated selection and input modules.

We operated our device in Dam-deficient bacteria since earlier results showed that natural *in vivo* Dam methylation may interfere with the Selection module's accurate synthetic hemi-methylation pattern (See Text S4, Table S2, Figure S3), which is essential for its strand selection activity.

We constructed the DNA device from a combination of natural and synthetic DNA components into a circular structure (Figure 1b) using a DNA editing technology previously developed by us [21]. We built the device with four functional components: (1) input and (2) Selection modules discussed earlier, (3) an origin of replication needed for device replication and (4) a Kanamycin resistance gene required for maintaining the device in the absence of Amp resistance (See sequence in Text S2).

The Selection module is a hemi-methylated dsDNA fragment composed of 2 partially overlapping ssDNA oligos 53 nt and 58 nt long (See selection module sequences: Text S2 and methylation optimization: Text S5, Figure S3). The module was designed to seamlessly integrate into the beginning of an Amp resistance gene coding region by altering its codon usage so that its non-looped strand does not disrupt Amp functionality, whereas the looped strand does. The location and orientation of the Amp gene within the device were designed so that the Selection module, embedded at the beginning of the Amp coding region, is less than 1 kb from the input module (See Figure 1c). This was crucial since the MMR system only processes mismatched DNA within 1 kb of the hemi-methylation site [14], [15].

In an earlier design the Amp gene had a dual function in our device both as the site of selection module integration and as a selection marker for device propagation. This generated a significant amount of false-positive colonies from devices that failed to integrate the Selection module but were resistant to Amp due to incomplete restriction cleavage of the selection module site. To eliminate this problem we modified the region of the Amp gene into which the selection module is integrated to encode a disrupted, non-functional version of it (that disrupts the Amp gene) and, at the same time, integrated a functional Kanamycin resistance gene into a different location on the device. This eliminated false positive colonies originating from inefficient Selection module integration and/or its incomplete restriction out of the device prior to its integration, while enabling propagation the device for preparative purposes using Kan selection. We added a control step for the proper digestion of the vector by the restriction enzymes (Text S6, Figure S4), optimized the sequences and concentrations in the ligation reaction (Text S7, Table S3, Figure S5), and validated that a synthetic loop is indeed largely invisible to the MMR system (Text S8, Figure S6).

We designed a controlled proof of concept experiment to test the device *in vivo* using a library of 38 erroneous GFP gene variants generated randomly by error-prone PCR and an additional variant for which we attempted to enrich (Text S9, Table S1). Each of these 39 variants was arbitrarily selected, did not code for a protein that confers any selective advantage and lacked any promoter that would result in its transcription, thereby eliminating any possible artifact enrichment (see full variant sequences in Text S2).

We controlled for two main features of our design and procedure (see elaborated experiment design: Text S10): (1) we controlled for the Selection modules strand selection activity using an inactivated version of the module which lacks the Amp disrupting loop structure and the two hemi-methylated sites and (2) for input module activity using an inactivated version of the module in which mismatches between input modules were not exposed by re-annealing (See Figure 3 and Table 1).

The ability of the device to enrich for a specific variant *in vivo* was evaluated by clone-sequencing the input module of devices from several hundred Dam-deficient bacterial clones (See Table 1) that were transformed with the device library. Enrichment was evaluated by observing deviations from the initial ratio of erroneous to correct input modules within the device library. We defined the enrichment factor of the fully activated device as follows: (Clones with correct input module/Clones with erroneous input module) divided by the same ratio from an identical control experiment with devices bearing inactivated Selection and input modules.

Results from these experiments show that a population of devices activated at both the Selection and input modules significantly enrich for devices with the correct input module compared to the negative control inactivated at both the Selection and input modules (See Figure 3 and Table 1, row 1). We further controlled for device features separately by transforming and clone sequencing the same input module population into (1) devices with inactivated input modules but with a functional Selection module and (2) devices with activated input modules but with inactivated Selection modules. Results from control (1) show that, as expected from our design, the Selection module cannot induce any statistically significant enrichment if the input module is inactivated compared with the fully inactivated device (See Figure 3 and Table 1, row 4). Nevertheless, we cannot rule out that any statistically insignificant enrichment observed (Table 1, row 4) may have resulted from the natural strand displacement rate of dsDNA even under the lack of deliberate re-annealing. Surprisingly, a second control with activated input modules and inactivated Selection modules did result in statistically significant enrichment (See Figure 3 and Table 1, row 2). The specific mechanism by which bacteria enrich for the correct sequence in this control is independent of the Selection module and depends on input module activation, possibly involving a mechanism for rejecting DNA that harbors mismatched DNA. It suggests a simple method for reducing the error rate in any DNA fragment prior to its cloning into bacteria by simply exposing its mismatched bases via re-annealing and transforming it to bacteria. The function of the Selection module was found to be significant by comparing the enrichment resulting from fully activated devices to devices with activated input modules but inactivated Selection modules (See Table 1, row 3).

Collectively, these results demonstrate that while synthetic devices with inactivated functional elements are largely non-functional, fully activated devices are active and result in significant enrichment for a specific input module.

## Discussion

MMR research has extensively studied the effect of MMR on various substrates both *in vivo* and *in vitro* [14]–[17], [26] elucidating its basic design principles. However, this knowledge of natural design principles has not been successfully utilized to engineer a functional synthetic device *in vivo* based on them.

Although our device is only modestly functional it presents an advance in the intricacy of the interface between engineered and endogenous cellular machinery and constitutes a step towards the development of an applied method for function independent DNA selection *in vivo*.

In contrast to assay-specific *in vivo* enrichment schemes developed so far in which enrichment is based on a particular catalytic activity selected for [26], [27], the design principles of our synthetic device enable the enrichment of any DNA sequence in bacteria regardless of any enzymatic or other function it may code for.

Further optimization of this prototype device, including bacterial strains besides the Dam mutant, loop structure, methylation method, device construction method and GATC depletion may improve its performance by providing a more seamless integration with the MMR system.

Future MMR interfacing devices, possibly with different design principles, may go beyond our proof-of-concept device and achieve enrichment capabilities that would make them applicable to various DNA enrichment related, “Needle in a haystack” problems in biology such as the detection of fetal DNA in maternal blood for the identification of various fetal abnormalities, blood circulating DNA markers of malignancies and for alleviating the problem of erroneous DNA in synthetic biology. To this end, the design of the system will be such that it will not select for the most abundant DNA molecule but for a predetermined molecule with a specific sequence. In this case, a complementary strand to the particular sequence being selected for will be annealed (in excess) to the molecules in the library, thereby enriching its fraction within the homoduplex population.

## Supporting Information

Figure S1 **In silico simulation of enrichment potential.** The initial fraction of devices with an error free input module (X axis) is plotted against the fraction of devices with an error free, homo-duplex input module out of the total population of homo-duplex devices. The curves (from right to left) represent increasing numbers of initial devices with erroneous DNA inputs. These graphs exemplify the fact that the enrichment factor of our system increases with library size.
(TIF)

Figure S2 **Comparison between the enrichment factor of W.T and Dam- strains.** Comparison between the enrichment factor of from two bacterial strains, E.cloni (Lucigen) and GM48, with and without a functional MMR system (Dam-), respectively. (A) Comparison between E. cloni enrichment factor negative control and operative device (colonies tested: n = 47 and n = 47 respectively). (B) GM48 enrichment factor comparison between negative control and a functional device (colonies tested: n = 92 and n = 80 respectively).
(TIF)

Figure S3 **Capillary electrophoresis analysis of digestion with MboI.** MboI is a restriction enzyme which digests dam sites (GATC) but is blocked by methylated and hemimethylated dam sites. We used MboI assay to test the methylation efficiency of in the Selection site (A) Digestion of dsDNA constructed of (1) the specially modified methylated strand, and (2) a complementary unmethylated oligonucleotide, labeled by HEX fluorophore. Results show that almost 100% of DNA molecules were not digested, implying that the methylated oligonucleotide is efficiently methylated; (B) Negative control - Digestion dsDNA constructed of (1) a standard unmethylated oligonucleotide, containing the same sequence as A1, and (2) the same labeled oligonucleotide as A2. Results show almost 100% digestions; (C) Digestion of a dsDNA containing sequences similar to A & B (2 dam sites), which was treated in vitro prior to the digestion by dam Methyltransferase (NEB) to create a fully methylated dsDNA. Results show poor protection by the methyl which was added during enzymatic reaction, indicating that chemical methylation is more efficient than enzymatic methylation.
(TIF)

Figure S4 **pIVEC (∼4000 bp) restriction enzymes control.** We have digested the vector with different restriction enzymes to verify their proper activity. Lane 1: undigested plasmid (supercoiled) lane 2: XhoI & SspdI (KasI), expected size ∼1050; lane 3: XhoI & HindIII, expected size ∼1000; lanes 4, 5 and 6: Single digestion using XhoI, SspdI (KasI) & HindIII respectively; lane 7: Digestion using SspdI (KasI) & HindIII (Actual enzymes which digest the Selection site during cloning experiment), expected size ∼50 bp.
(TIF)

Figure S5 **Colony count results.** (A) Series name indicates Selection site: Vector ratio in ligation reaction and x-axis determine the type of Selection site structure (Table S3); (B) Transformation results of supercoiled amp+ & amp− plasmids as positive & negative controls respectively. Results are normalized according to: (Colonies formed on LB Kanamycin50+Ampicilin200)/(Colonies formed on LB Kanamycin).
(TIF)

Figure S6 **Example of sequencing analysis of a colony containing two different DNA molecules.** Two kinds of populations are detectable by sequencing the plasmid from one direction: A division to two kinds of sequences is exhibited at the start location of the loop structure (heteroduplex DNA)..
(TIF)

Table S1 **Mutation analysis of GFP variants, produced using error-prone PCR, compared with error-free GFP sequence reference.**
(PDF)

Table S2 **Selection sites structures which serve as controls for experiments and their expected result after transformation to E. coli.** (A) positive control - restores the ampicillin resistance, bacteria should live; (B) negative control - inserts a frame shift to β-lactamase gene, bacteria should die; (C) the tested system with a functional selection site, bacteria should live only if no mismatch was found (See Figure 1c).
(TIF)

Table S3 **Selection sites structures and their expected viability decision, in transformed** ***E. coli*** **.**
(TIF)

Text S1 **Supplementary methods.**
(DOC)

Text S2 **Supplementary sequences.**
(DOC)

Text S3 ***In silico*** **simulation of enrichment potential.**
(DOC)

Text S4 **Device operation in wild type Vs. Dam deficient bacteria -** ***In vivo*** **Dam methylation may reprogram the device.**
(DOC)

Text S5 **Methylation evaluation experiment - Selection module hemi-methylation is highly efficient.**
(DOC)

Text S6 **Restriction control for the insertion of the Selection module.**
(DOC)

Text S7 **Selection module loop optimization - Integration of the Selection module loop into the device can be optimized.**
(DOC)

Text S8 **Invisibility of the selection module loop to the MMR system -The selection module loop is partially invisible to the MMR system.**
(DOC)

Text S9 **Generating the library of input modules using error-prone PCR.**
(DOC)

Text S10 **Description of experimental procedures.**
(DOC)
