# Correction: Programmable In Vivo Selection of Arbitrary DNA Sequences

**DOI:** 10.1371/annotation/8e888220-8d00-4552-b468-4694e6bd9d47

**Published:** 2012-11-19

**Authors:** Tuval Ben Yehezkel, Tamir Biezuner, Gregory Linshiz, Yair Mazor, Ehud Shapiro

The Author Contributions are incorrect. They should be: Conceived the experiments: TBY GL. Designed the experiments: TB TBY GL. Performed the experiments: TB GL TBY. Analyzed the data: TB TBY GL YM. Wrote the paper: TBY TB ES.

