# Supplementary figures and images for: Programmable In Vivo Selection of Arbitrary DNA Sequences
Source: PLoS One. 2012 Nov 14;7(11):e47795. doi: 10.1371/journal.pone.0047795 (PMC3498277; doi:10.1371/journal.pone.0047795)

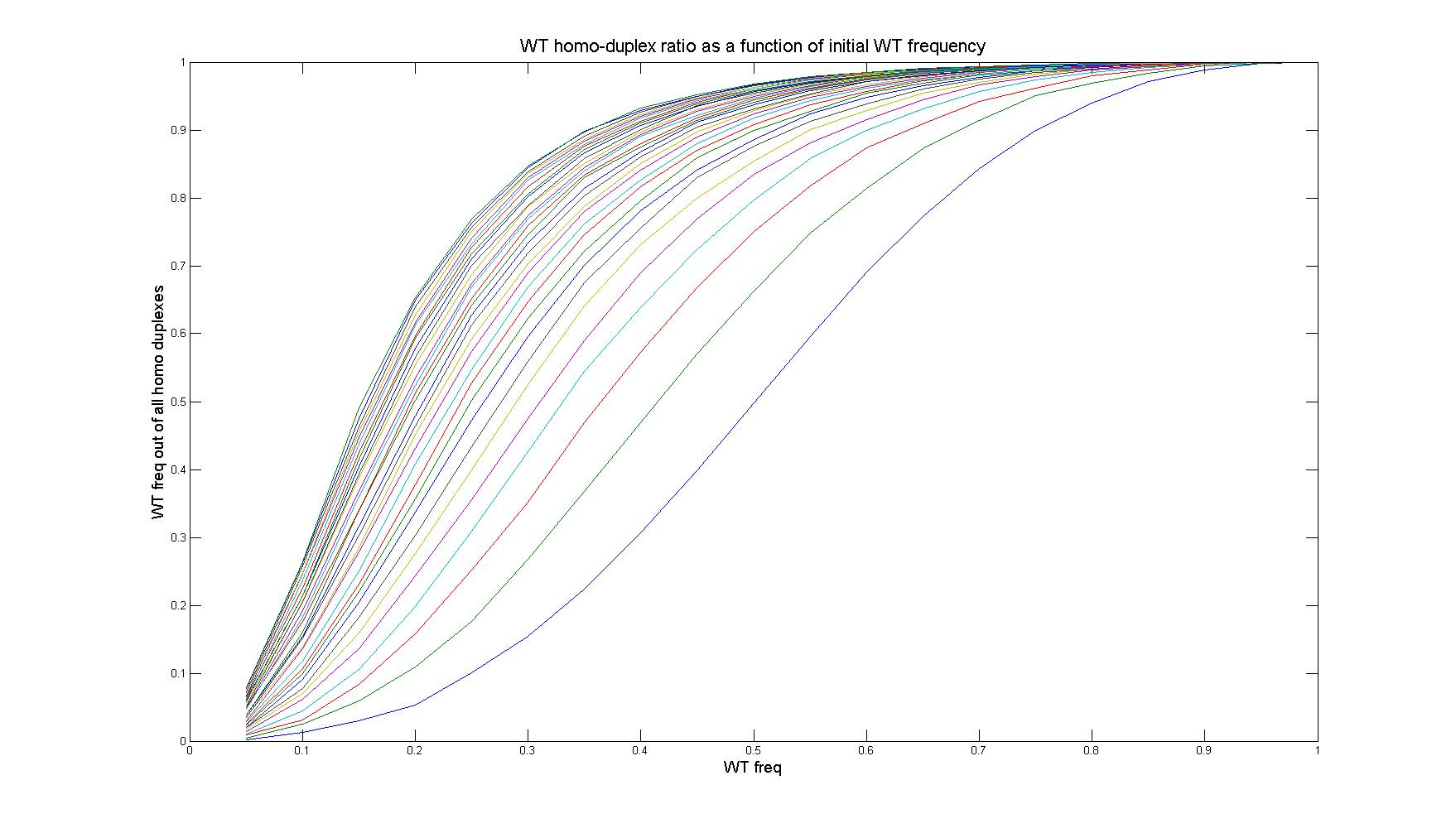

Supplement: Figure S1 — In silico simulation of enrichment potential. The initial fraction of devices with an error free input module (X axis) is plotted against the fraction of devices with an error free, homo-duplex input module out of the total population of homo-duplex devices. The curves (from right to left) represent increasing numbers of initial devices with erroneous DNA inputs. These graphs exemplify the fact that the enrichment factor of our system increases with library size. (TIF) [file pone.0047795.s001.tif]

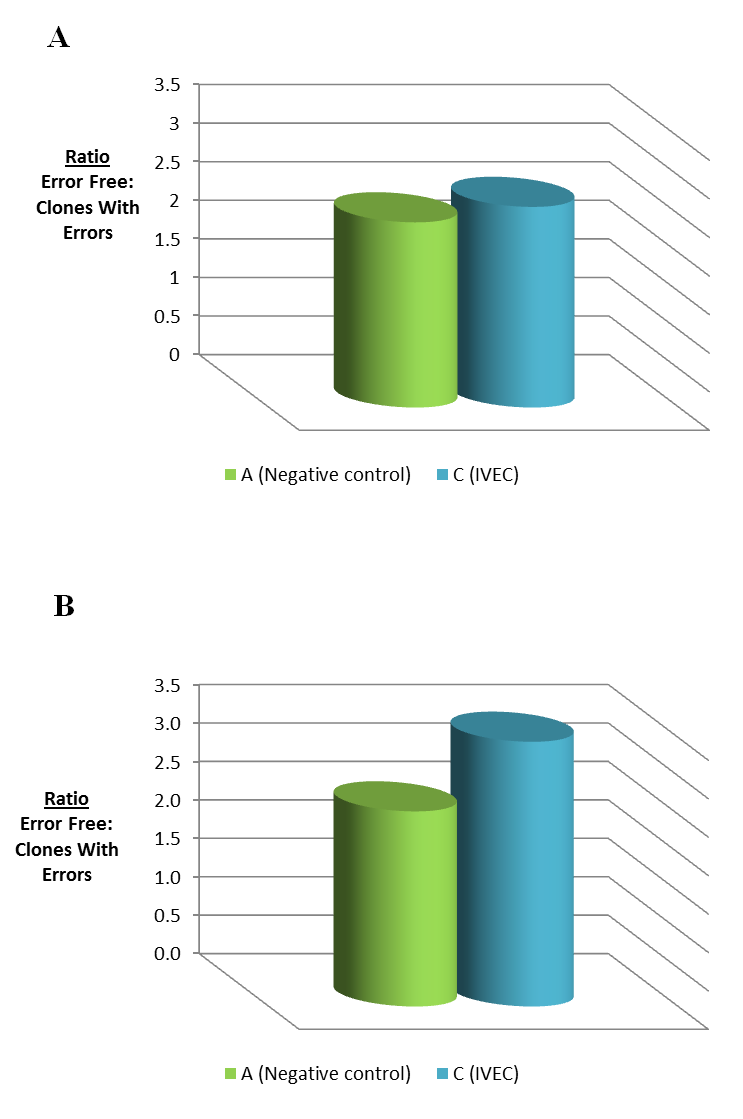

Supplement: Figure S2 — Comparison between the enrichment factor of W.T and Dam- strains. Comparison between the enrichment factor of from two bacterial strains, E.cloni (Lucigen) and GM48, with and without a functional MMR system (Dam-), respectively. (A) Comparison between E. cloni enrichment factor negative control and operative device (colonies tested: n = 47 and n = 47 respectively). (B) GM48 enrichment factor comparison between negative control and a functional device (colonies tested: n = 92 and n = 80 respectively). (TIF) [file pone.0047795.s002.tif]

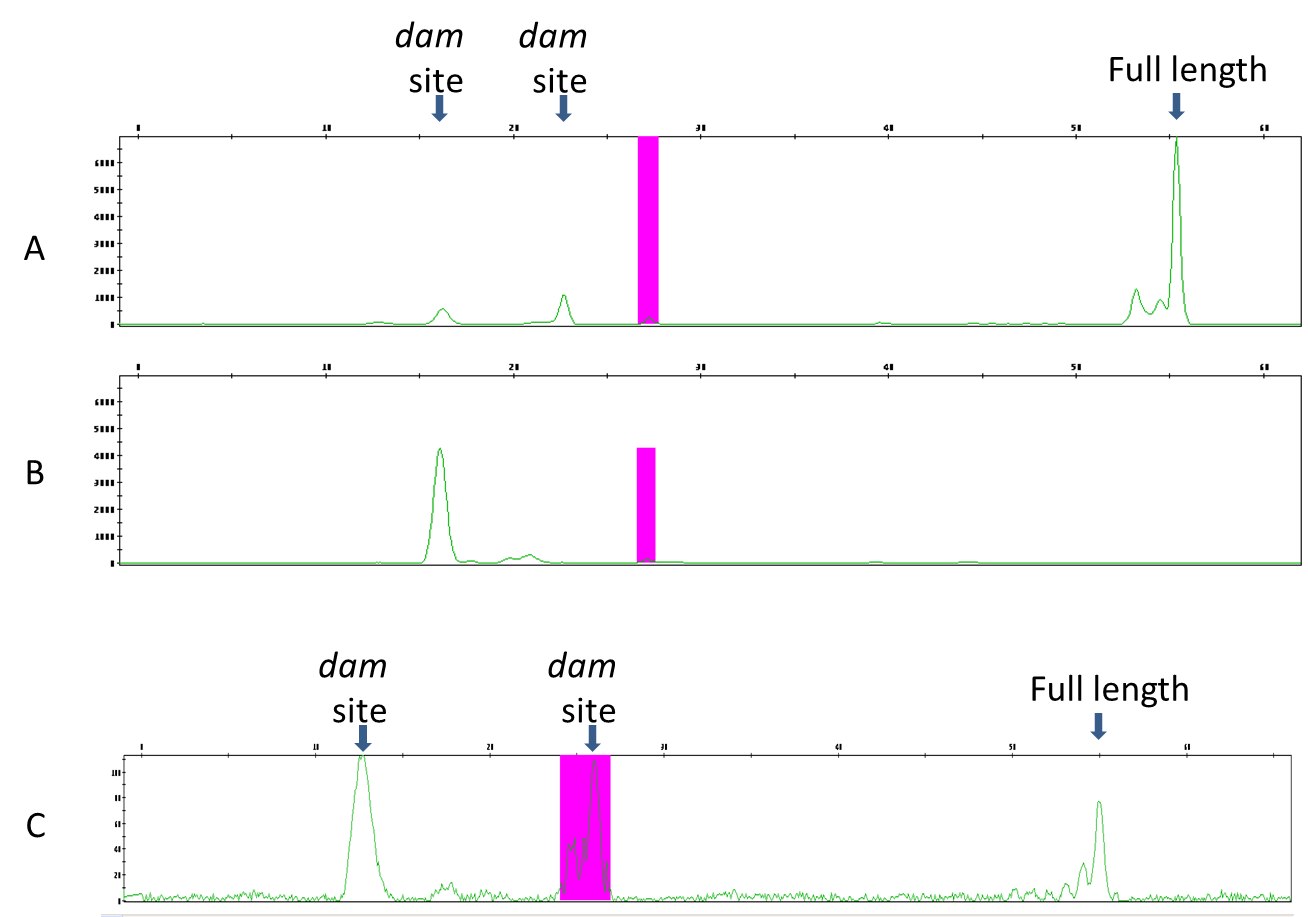

Supplement: Figure S3 — Capillary electrophoresis analysis of digestion with MboI. MboI is a restriction enzyme which digests dam sites (GATC) but is blocked by methylated and hemimethylated dam sites. We used MboI assay to test the methylation efficiency of in the Selection site (A) Digestion of dsDNA constructed of (1) the specially modified methylated strand, and (2) a complementary unmethylated oligonucleotide, labeled by HEX fluorophore. Results show that almost 100% of DNA molecules were not digested, implying that the methylated oligonucleotide is efficiently methylated; (B) Negative control - Digestion dsDNA constructed of (1) a standard unmethylated oligonucleotide, containing the same sequence as A1, and (2) the same labeled oligonucleotide as A2. Results show almost 100% digestions; (C) Digestion of a dsDNA containing sequences similar to A & B (2 dam sites), which was treated in vitro prior to the digestion by dam Methyltransferase (NEB) to create a fully methylated dsDNA. Results show poor protection by the methyl which was added during enzymatic reaction, indicating that chemical methylation is more efficient than enzymatic methylation. (TIF) [file pone.0047795.s003.tif]

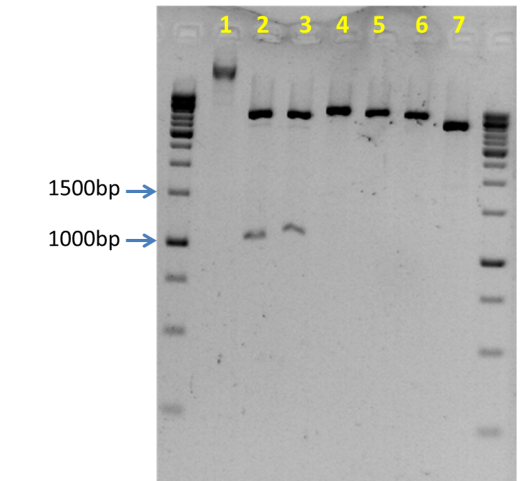

Supplement: Figure S4 — pIVEC (∼4000 bp) restriction enzymes control. We have digested the vector with different restriction enzymes to verify their proper activity. Lane 1: undigested plasmid (supercoiled) lane 2: XhoI & SspdI (KasI), expected size ∼1050; lane 3: XhoI & HindIII, expected size ∼1000; lanes 4, 5 and 6: Single digestion using XhoI, SspdI (KasI) & HindIII respectively; lane 7: Digestion using SspdI (KasI) & HindIII (Actual enzymes which digest the Selection site during cloning experiment), expected size ∼50 bp. (TIF) [file pone.0047795.s004.tif]

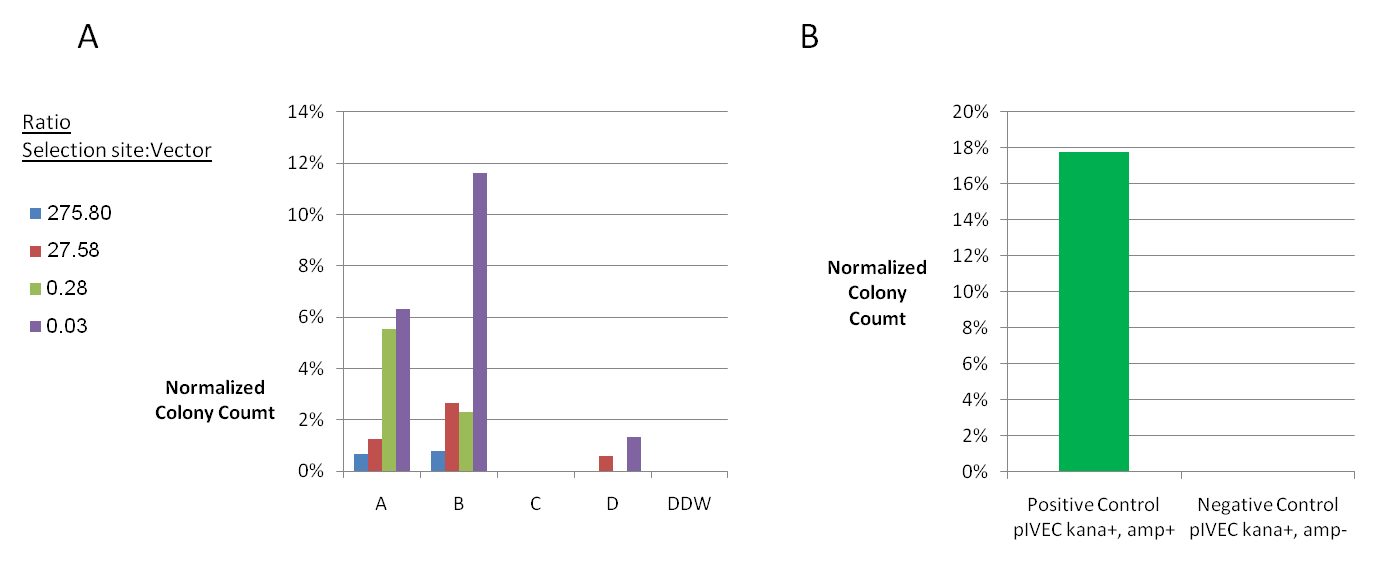

Supplement: Figure S5 — Colony count results. (A) Series name indicates Selection site: Vector ratio in ligation reaction and x-axis determine the type of Selection site structure (Table S3); (B) Transformation results of supercoiled amp+ & amp− plasmids as positive & negative controls respectively. Results are normalized according to: (Colonies formed on LB Kanamycin50+Ampicilin200)/(Colonies formed on LB Kanamycin). (TIF) [file pone.0047795.s005.tif]

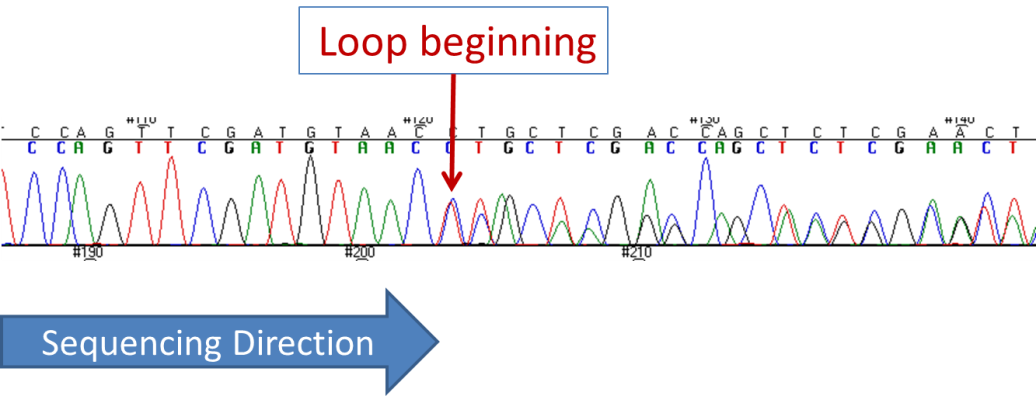

Supplement: Figure S6 — Example of sequencing analysis of a colony containing two different DNA molecules. Two kinds of populations are detectable by sequencing the plasmid from one direction: A division to two kinds of sequences is exhibited at the start location of the loop structure (heteroduplex DNA).. (TIF) [file pone.0047795.s006.tif]

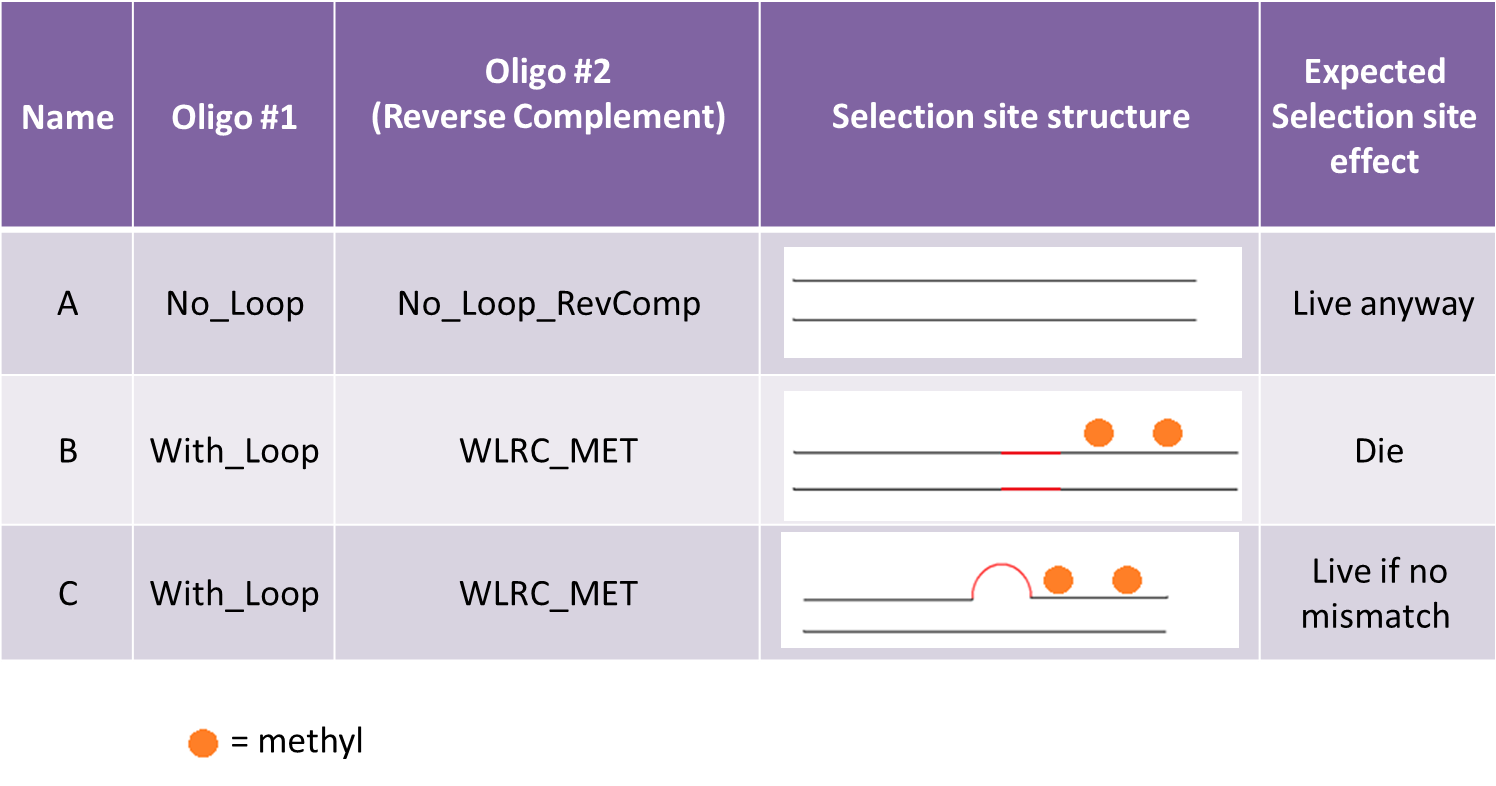

Supplement: Table S2 — Selection sites structures which serve as controls for experiments and their expected result after transformation to E. coli. (A) positive control - restores the ampicillin resistance, bacteria should live; (B) negative control - inserts a frame shift to β-lactamase gene, bacteria should die; (C) the tested system with a functional selection site, bacteria should live only if no mismatch was found (See Figure 1c). (TIF) [file pone.0047795.s008.tif]

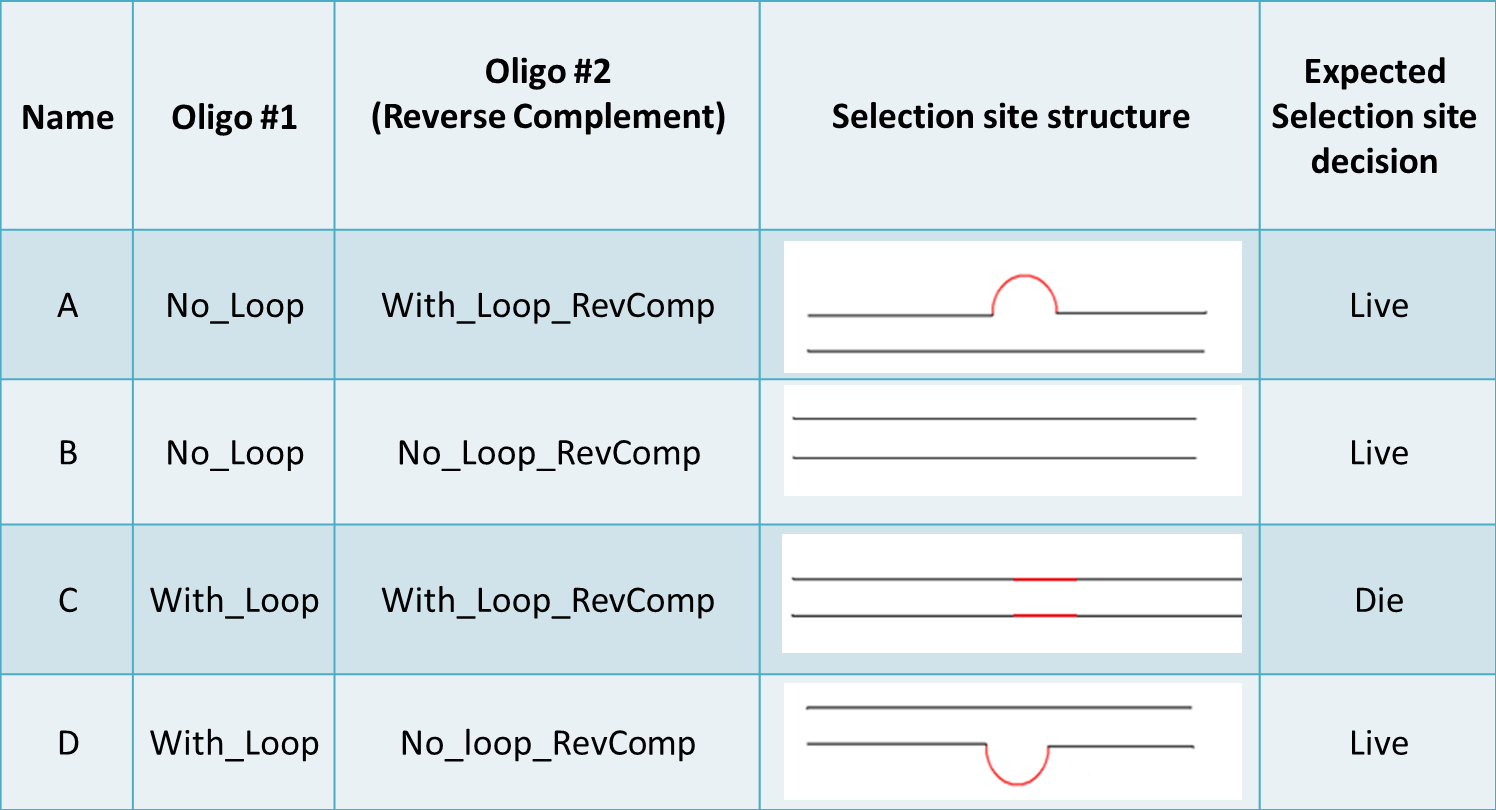

Supplement: Table S3 — Selection sites structures and their expected viability decision, in transformed E. coli . (TIF) [file pone.0047795.s009.tif]
