# Supplementary material for: Programmable In Vivo Selection of Arbitrary DNA Sequences
Source: PLoS One. 2012 Nov 14;7(11):e47795. doi: 10.1371/journal.pone.0047795 (PMC3498277; doi:10.1371/journal.pone.0047795)
Supplement: Table S1 — Mutation analysis of GFP variants, produced using error-prone PCR, compared with error-free GFP sequence reference. (PDF) [file pone.0047795.s007.pdf]

Variance Table Report

For research purposes only. Not intended for clinical use.

Date: March 10, 2011
Project Name: 45 GFP screening.SPF
Compare Consensus to Reference: GFP_23112009
Comparison Range: Unfiltered
Base Positions: 1 to 762
Options: Large gap insertions (10 or more bases) included.
Matches to ambiguous reference positions included.

Reference		E10	E13	E14	E15	E1	E16	E17	E18	E19	E2	E21	E22	E23	E24	E25	E26	E27	E28	E3	E30	E31	E32	E34
9	C																						A	
16	C				A																			
24	T																							
25	G							A																
29	A												T											
32	G				A																			
39	A				T																			
60	C				T																			
63	T				C																			
72	A								T															
85	C							T																
89	C							:																
92	A																							
95	T																	A						
106	C																							
108	G					A																		
118	G																			A				
119	G																				A			
126	C													A										
139	G																							A
145	C																							
146	C			A																				
150	A																							
157	C																T							
162	C																				T			
167	G												A											
170	A																G							
182	C																		T					
183	C							T																
184	C																							
187	G																							
200	A									G														
202	C																							
207	T																C							
217	C																							
220	G				A																			
221	C																					A		
228	T																		A					
242	G				T																			
243	A								G															

248	A																							
251	A																							
263	T								A															
264	T																							
267	T									A														
268	C																	T						
269	A																	T						
281	C				T																			
286	A																			T				
289	C	A																						
293	G							A																
303	G	A																						
304	C											T												
305	A															T								
311	T																							
312	T																						C	
316	C											T												
317	A																							
333	A																					G		
339	C															G								
342	G																	A						
347	G						T																	
354	A							T																
372	T								C															
374	G																							
384	T																			C				
391	G																							
396	G																							
407	A															T								
408	A								T															
416	G											T												
417	G									T														
421	C		A																					
431	C														T									
436	G																T							
438	T								C															
445	C								T															
446	A																							
450	A													T										
453	A																							
454	C			T																				
456	G				A																A			
460	C		T																					
464	G																							
470	A				G																			
472	C																			A				
478	C			A																				
486	A			G																				
489	A																							
495	G																							A
506	A						T																	
507	A																							
519	G														A									
528	T										A													
529	C																							

530	G												A											
532	G																							
543	T																			A				
544	G																							
549	T														C									
555	A																			T				
556	C																							
559	C					T																		
563	C																				T			
566	C																							
568	G																							
572	A										G													
574	C																							
577	C			T																				
602	C																							
618	T															C								
622	C																							
623	A		C																					
624	C																							
635.1	:																							
636	T											C												
638	A																						G	
652	C			T																				
653	G												A											
657	A	T																						
667	C																		T					
671	G																			:				
683	T							A																
689	A						G																	
693	C					T																		
694	C																							
702	T																							
703	C						G																	
705	C																					G		
706	T																							
717	A																G							
721	G																					A		
722	C													T										
726	A																							
728	A			T									T											
746	T																							C
750	G											T												
757	A																		G					
758	T							A																
Total		3	3	7	10	3	4	8	7	3	2	5	5	3	3	4	5	4	4	7	4	4	3	3

E35	E36	E37	E38	E39	E4	E40	E41	E42	E44	E5	E6	E7	E8	E9	Total
															1
			A												2
				C											1
															1
															1
															1
															1
															1
															1
															1
															1
									A						2
														G	1
															1
	T														1
															1
															1
															1
															1
															1
													A		1
															1
												T			1
														A	2
															1
															1
															1
															1
															1
	A														1
			A												1
															1
A															1
															1
						A					T				2
															1
															1
															1
									T						2
															1
														T	1
C															1
															1
			C												1
								C							2
															1
															1
															1
															1
															1
															1
															1
															1
															1

		A													1
									A				A		2
															1
										G					1
															1
															1
															1
															1
															1
															1
		A													1
															1
	A														1
		T													1
															1
															1
															1
															1
									T						2
															1
															1
															1
															1
		T													1
															1
														T	1
															1
															2
															1
														A	1
		C					T								3
															1
															1
															1
													G		1
															1
															1
									G						1
															1
															1
								A							1
															1
										T					1
															1
											A				1
															1
															1
		T													1
															1
															1
											T				1
													A		1
															1
								T							1
															1

	T														1
															1
G															1
															1
			T												1
													C		1
															1
															1
															1
															1
															1
															1
															1
															1
															1
		T													2
				T											1
					A										1
															1
															1
	A														1
															1
															1
							T								2
							T								1
															2
															1
															1
															1
															1
3	5	7	4	2	1	1	3	3	5	2	3	1	4	5	153

Sequence Name	Comparison Range Coverage	
E10	Complete	1 to 762
E13	Complete	1 to 762
E14	Complete	1 to 762
E15	Complete	1 to 762
E1	Complete	1 to 762
E16	Complete	1 to 762
E17	Complete	1 to 762
E18	Complete	1 to 762
E19	Complete	1 to 762
E2	Complete	1 to 762
E21	Complete	1 to 762
E22	Complete	1 to 762
E23	Complete	1 to 762
E24	Complete	1 to 762
E25	Complete	1 to 762
E26	Complete	1 to 762
E27	Complete	1 to 762
E28	Complete	1 to 762
E3	Complete	1 to 762
E30	Complete	1 to 762
E31	Complete	1 to 762

E32	Complete	1 to 762
E34	Complete	1 to 762
E35	Complete	1 to 762
E36	Complete	1 to 762
E37	Complete	1 to 762
E38	Complete	1 to 762
E39	Complete	1 to 762
E4	Complete	1 to 762
E40	Complete	1 to 762
E41	Complete	1 to 762
E42	Complete	1 to 762
E44	Complete	1 to 762
E5	Complete	1 to 762
E6	Complete	1 to 762
E7	Complete	1 to 762
E8	Complete	1 to 762
E9	Complete	1 to 762

Gene Codes Corporation TCA GENE AGT CODES *Report generated by Sequencher™: The complete software solution for sequencing DNA.*
