# Supplementary material for: Programmable In Vivo Selection of Arbitrary DNA Sequences
Source: PLoS One. 2012 Nov 14;7(11):e47795. doi: 10.1371/journal.pone.0047795 (PMC3498277; doi:10.1371/journal.pone.0047795)
Supplement: Text S1 — Supplementary methods. (DOC) [file pone.0047795.s010.doc]

**Supplementary methods**

**Mixes Preparation**

Premade mixes for Phosphorylation, Elongation, PCR and Lambda Exonuclease Digestion (including enzymes & buffers) were mixed at X5 concentration and added to reaction immediately before the reaction.

**Plasmid Construction**

pIVEC (see sequence in Text S2), the plasmid which was constructed for the IVEC system was planned according to our software and algorithms and was constructed according to our DNA construction method .

**Phosphorylation**

300pmol of single stranded DNA in a 50 µl reaction containing 70 mM Tris-HCl, 10mM MgCl2, 7 mM dithiothreitol, pH 7.6 at 37°C, 1 mM ATP & 10 units T4 Polynucleotide Kinase (NEB). Reaction is incubated at 37°C for 30 min, then at 42°C for 10 min and inactivated at 65°C for 20 min.

**Elongation**

1pmol of single stranded DNA in a 25µl reaction containing 5µl AccuSure DNA Polymerase buffer X10 (Supplied with enzyme), 250µM of each of dNTPs, 5U of AccuSure DNA Polymerase (BioLine) and SYBR Green diluted 1:50,000.

Thermal Cycler program is: Enzyme activation at 95°C for 10 min (activation of AccuSure), followed by 3 cycles of: 95oc 1 sec, 50oc 5 min (cooling 0.1oc/sec), elongation at 72oc for 10 min (heating 0.1oc/sec).

**PCR**

Most standard PCR reactions were done according to the following protocol unless mentioned otherwise: 1-0.1fmol template, 10pmol of each primer in a 32.5 µl reaction containing 250µM of each of dNTPs, AccuSure DNA Polymerase buffer X10 (Supplied with enzyme), 2.43U of AccuSure DNA Polymerase (BioLine) and SYBR Green diluted 1:50,000.

Thermal Cycler program is: Enzyme activation at 95°C 10 min. 20-30 cycles of: Denaturation 95°C for 20sec, annealing at Tm of primers for 30sec, extension 72°C for 1-1:30 min per kbp of template. A final elongation was performed at 72°C for 1:30 min.

**Lambda Exonuclease digestion**

1-5pmol of 5' phosphorylated DNA termini in a 30µl reaction containing 67mM Glycine-KOH, 2.5mM MgCl2, 0.01% Triton X-100, 4mM Dithiothreitol, 5.5U Lambda Exonuclease (Epicentre) and SYBR Green diluted 1:50,000.

Thermal Cycler program is 30°C for 15 min, 42°C for 10 min, enzyme inactivation at 65°C for 10 min.

**Automatic liquid handling**

Most of the reactions done in a 96-well plate were performed by automatic liquid handling robots: Freedom 2000 & Freedom EVO 200 (TECAN), controlled by in-house developed software.

**Capillary Electrophoresis**

2µl dsDNA molecules with one 5' fluorescently labeled oligonucleotides with HEX fluorophore were diluted in a 10µl formamide solution containing GeneScan 500 LIZ size marker, diluted 1:40 (ABI). Fragment analysis was done on an ABI 3130 genetic Analyzer.

**Selection module design and construction**

The hetero-duplex Selection module was generated from synthetic oligonucleotides containing several elements: (1) a 5nt loop, comprised of a stop codon and 2 frame-shifting bases, which ensure disruption of the Amp gene, (2) two adjacent hemimethylated GATC (Dam) sites that were inserted using synonymous codons and (3) two flanking cohesive sspDI and HindIII ends for ligating the Selection module into the device which were also inserted using synonymous codons.

**Oligonucleotide Annealing**

10 pmol of each oligonucleotide were mixed with AccuSure DNA Polymerase buffer X10 (BioLine) in a 20µl reaction. Thermal Cycler program is Denaturation at 95°C for 2 min, followed by slow cooling 0.1oc/sec to 4°C.

**Vector digestion**

Digestion of vector was done according to the recommended enzyme ratios according to the manufacture guidelines for double digestion (HindIII and sspDI). Digestion protocol was as follows: 90minutes of Digestion in 37°C. Digestion product was later purified using MinElute (Qiagen).

**Ligation**

Ligation was done using T4-Ligase (Fermentas) using the manufacturers standard protocol. Specifically, annealed oligos bearing cohesive ends (HindIII and sspDI) were diluted 1:100 and 20-30 femtomoles were ligated with 25ng double digested vector (also HindIII and sspDI). The ligation product was purified using MinElute (Qiagen).

**Cloning strain**

For standard cloning, the E. coli strain which was used is a chemically modified JM109 strain. Cloning was done using standard procedure (Promega).

For electroporation cloning, the E. coli strains which were used are: E. cloni (Lucigen) and GM48 (dam-, dcm-), courtesy of Prof. Zvi Livneh's laboratory.

**Chemically Modified Competent Cells**

Chemically modified competent cells were prepared according to protocol by Sambrook *et al.* .

**Electrocompetent Cells Preparation**

Fresh bacterial colony was picked to inoculate a "starter" containing LB, which was grown O.N at 37°C. 5ml from starter were then mixed with 1L LB which was grown until reaching 0.5-0.8 OD. A washing step which included: Centrifugation, supernatant disposal and resuspension using sterile DDW, was repeated 3 times. After another washing step, the final pellet was resuspended using 4-10 ml of 10% glycerol, followed by aliquots distribution, snap freeze in liquid nitrogen and storage at -80oC.

**Colony PCR**

A single colony was picked with a tip and inoculation of the colony in a petri dish containing a grid was performed. The same tip was placed for at least 15 min in a 200µl DDW. 2µl from the inoculated DDW were used as a template for a 10µl PCR reaction which contained 4pmol of each primer, 250µM of each of dNTPs, AccuSure DNA Polymerase buffer X10 (Supplied with enzyme), 0.75U of AccuSure DNA Polymerase (BioLine) and SYBR Green diluted 1:50,000.

Thermal Cycler program is: Enzyme activation at 95°C 10 min. 27-30 cycles of: Denaturation 95°C for 30sec, annealing at 60°C of primers for 30sec and extension 72°C for 1:52 min. A melt-curve step is followed: Denaturation at 95°C and slow cooling at 0.1oc/sec to 37°C.

**Supplementary references**

1. Linshiz G, Ben Yehezkel T, Kaplan S, Gronau I, Ravid S, et al. (2008) Recursive construction of perfect DNA molecules from imperfect oligonucleotides. Molecular Systems Biology: -.

2. Shabi U, Kaplan S, Linshiz G, Benyehezkel T, Buaron H, et al. (2010) Processing DNA molecules as text. Syst Synth Biol 4: 227-236.

3. Sambrook J, Russell DW, Janssen K, Argentine J, Cold Spring Harbor L (2001) Molecular cloning a laboratory manual on the web. Cold Spring Harbor, N.Y.: Cold Spring Harbor Laboratory.
