# Supplementary material for: Programmable In Vivo Selection of Arbitrary DNA Sequences
Source: PLoS One. 2012 Nov 14;7(11):e47795. doi: 10.1371/journal.pone.0047795 (PMC3498277; doi:10.1371/journal.pone.0047795)
Supplement: Text S2 — Supplementary sequences. (DOC) [file pone.0047795.s011.doc]

**Supplementary sequences**

The origin of replication of pIVEC was left unchanged from that of pGEM. The kanamycin resistance gene was incorporated from pRSFDuet (Novagen) with a codon change, to eliminate a system essential restriction site.

>pIVEC_GFP_with_loop

CAAACAAACCACCGCTGGTAGCGGTGGTTTTTTTGTTTGCAAGCAGCAGATTACGCGCAGAAAAAAAGGATCTCAAGAAGATCCTTTGATCTTTTCTACGGGGTCTGACGCTCAGTGGAACGAAAACTCACGTTAAGGGATTTTGGTCATGAGATTATCAAAAAGGATCTTCACCTAGATCCTTTTAAATTAAAAATGAAGTTTTAAATCAATCTAAAGTATATATGAGTAAACTTGGTCTGACAGTTACCAATGCTTAATCAGTGAGGCACCTATCTCAGCGATCTGTCTATTTCGTTCATCCATAGTTGCCTGACTCCCCGTCGTGTAGATAACTACGATACGGGAGGGCTTACCATCTGGCCCCAGTGCTGCAATGATACCGCGAGACCCACGCTCACCGGCTCCAGATTTATCAGCAATAAACCAGCCAGCCGGAAGGGCCGAGCGCAGAAGTGGTCCTGCAACTTTATCCGCCTCCATCCAGTCTATTAATTGTTGCCGGGAAGCTAGAGTAAGTAGTTCGCCAGTTAATAGTTTGCGCAACGTTGTTGCCATTGCTACAGGCATCGTGGTGTCACGCTCGTCGTTTGGTATGGCTTCATTCAGCTCCGGTTCCCAACGATCAAGGCGAGTTACATGATCCCCCATGTTGTGCAAAAAAGCGGTTAGCTCCTTCGGTCCTCCGATCGTTGTCAGAAGTAAGTTGGCCGCAGTGTTATCACTCATGGTTATGGCAGCACTGCATAATTCTCTTACTGTCATGCCATCCGTAAGATGCTTTTCTGTGACTGGTGAGTACTCAACCAAGTCATTCTGAGAATAGTGTATGCGGCGACCGAGTTGCTCTTGCCCGGCGTCAATACGGGATAATACCGCGCCACATAGCAGAACTTTAAAAGTGCTCATCATTGGAAAACGTTCTTCGGGGCGAAAGCTTTCAAGGATCTTACCGCTGTTGAGATCCAGTTCGATGTAACTTACTCCACTCGGGCGCCCAACTGATCTTCAGCATCTTTTACTTTCACCAGCGTTTCTGGGTGAGCAAAAACAGGAAGGCAAAATGCCGCAAAAAAGGGAATAAGGGCGACACGGAAATGTTGAATACTCATACTCTTCCTTTTTCAATATTATTGAAGCATTTATCAGGGTTATTGTCTCATGAGCGGATACATATTTGAATGTATTTAGAAAAATAAACAAATAGGGGTTCCGCGCACCCCGGGGCTGATTATGATCTAGAGTCGCGGCCGCTTTACTTGTACAGCTCGTCCATGCCGAGAGTGATCCCGGCGGCGGTCACGAACTCCAGCAGGACCATGTGACCGCGCTTCTCGTTGGGGTCTTTGCTCAGGGCGGACTGGGTGCTCAGGTAGTGGTTGTCGGGCAGCAGCACGGGGCCGTCGCCGATGGGGGTGTTCTGCTGGTAGTGGTCGGCGAGCTGCACGCTGCCGTCCTCGATGTTGTGGCGGATCTTGAAGTTCACCTTGATGCCGTTCTTCTGCTTGTCGGCCATGATATAGACGTTGTGGCTGTTGTAGTTGTACTCCAGCTTGTGCCCCAGGATGTTGCCGTCCTCCTTGAAGTCGATGCCCTTCAGCTCGATGCGGTTCACCAGGGTGTCGCCCTCGAACTTCACCTCGGCGCGGGTCTTGTAGTTGCCGTCGTCCTTGAAGAAGATGGTGCGCTCCTGGACGTAGCCTTCGGGCATGGCGGACTTGAAGAAGTCGTGCTGCTTCATGTGGTCGGGGTAGCGGCTGAAGCACTGCACGCCGTAGGTCAGGGTGGTCACGAGGGTGGGCCAGGGCACGGGCAGCTTGCCGGTGGTGCAGATGAACTTCAGGGTCAGCTTGCCGTAGGTGGCATCGCCCCCGCCCTCGCCGGACACGCTGAACTTGTGGCCGTTTATGTCGCCGTCGACCAGGATGGGCACCACCCCAGTGAACAGCTCCTCGCCCTTGCTCACCATGGTGGCGACCGGTGGATCCCTCGAGGCCAGTGAATTGTAATACGACTCACTATAGGGCGAATTGGGCCCGACGTCGCATGCTCCCGGCCGCCAT::G:GCGGC:CGCGG:GA:ATTCGATTATCACTAGTTACATTCAAATATGTATCCGCTCATGAATTAATTCTTAGAAAAACTCATCGAGCATCAAATGAAACTGCAATTTATTCATATCAGGATTATCAATACCATATTTTTGAAAAAGCCGTTTCTGTAATGAAGGAGAAAACTCACCGAGGCAGTTCCATAGGATGGCAAGATCCTGGTATCGGTCTGCGATTCCGACTCGTCCAACATCAATACAACCTATTAATTTCCCCTCGTCAAAAATAAGGTTATCAAGTGAGAAATCACCATGAGTGACGACTGAATCCGGTGAGAATGGCAAAAGTTTATGCATTTCTTTCCAGACTTGTTCAACAGGCCAGCCATTACGCTCGTCATCAAAATCACTCGCATCAACCAAACCGTTATTCATTCGTGATTGCGCCTGAGCGAGACGAAATACGCGGTCGCTGTTAAAAGGACAATTACAAACAGGAATCGAATGCAACCGGCGCAGGAACACTGCCAGCGCATCAACAATATTTTCACCTGAATCAGGATATTCTTCTAATACCTGGAATGCTGTTTTCCCTGGGATCGCAGTGGTGAGTAACCATGCATCATCAGGAGTACGGATAAAATGCTTGATGGTCGGAAGAGGCATAAATTCCGTCAGCCAGTTTAGTCTGACCATCTCATCTGTAACATCATTGGCAACGCTACCTTTGCCATGTTTCAGAAACAACTCTGGCGCATCGGGCTTCCCATACAATCGATAGATTGTCGCACCTGATTGCCCGACATTATCGCGAGCCCATTTATACCCATATAAATCAGCATCCATGTTGGAATTTAATCGCGGCCTAGAGCAAGACGTTTCCCGTTGAATATGGCTCATACTCTTCCTTTTTCAATATTATTGAAGCATTTATCAGGGTTATTGTCTCATGAGCGGATACATATTTGAATGTAACTAGTGAATTCGCGGCCGCCTGCAGGTCGACCATATGGGAGAGCTCCCAACGCGTTGGATGCATAGCTTGAGTATTCTATAGTGTCACCTAAATAGCTTGGCGTAATCATGGTCATAGCTGTTTCCTGTGTGAAATTGTTATCCGCTCACAATTCCACACAACATACGAGCCGGAAGCATAAAGTGTAAAGCCTGGGGTGCCTAATGAGTGAGCTAACTCACATTAATTGCGTTGCGCTCACTGCCCGCTTTCCAGTCGGGAAACCTGTCGTGCCAGCTGCATTAATGAATCGGCCAACGCGCGGGGAGAGGCGGTTTGCGTATTGGGCGCTCTTCCGCTTCCTCGCTCACTGACTCGCTGCGCTCGGTCGTTCGGCTGCGGCGAGCGGTATCAGCTCACTCAAAGGCGGTAATACGGTTATCCACAGAATCAGGGGATAACGCAGGAAAGAACATGTGAGCAAAAGGCCAGCAAAAGGCCAGGAACCGTAAAAAGGCCGCGTTGCTGGCGTTTTTCCATAGGCTCCGCCCCCCTGACGAGCATCACAAAAATCGACGCTCAAGTCAGAGGTGGCGAAACCCGACAGGACTATAAAGATACCAGGCGTTTCCCCCTGGAAGCTCCCTCGTGCGCTCTCCTGTTCCGACCCTGCCGCTTACCGGATACCTGTCCGCCTTTCTCCCTTCGGGAAGCGTGGCGCTTTCTCATAGCTCACGCTGTAGGTATCTCAGTTCGGTGTAGGTCGTTCGCTCCAAGCTGGGCTGTGTGCACGAACCCCCCGTTCAGCCCGACCGCTGCGCCTTATCCGGTAACTATCGTCTTGAGTCCAACCCGGTAAGACACGACTTATCGCCACTGGCAGCAGCCACTGGTAACAGGATTAGCAGAGCGAGGTATGTAGGCGGTGCTACAGAGTTCTTGAAGTGGTGGCCTAACTACGGCTACACTAGAAGAACAGTATTTGGTATCTGCGCTCTGCTGAAGCCAGTTACCTTCGGAAAAAGAGTTGGTAGCTCTTGATCCGG

Color code:

Blue – Selection module (activated)

Green – Input module (GFP)

Grey – Kan resistance gene

>GFP_w.t

GGATCCACCGGTCGCCACCATGGTGAGCAAGGGCGAGGAGCTGTTCACTG

GGGTGGTGCCCATCCTGGTCGACGGCGACATAAACGGCCACAAGTTCAGC

GTGTCCGGCGAGGGCGGGGGCGATGCCACCTACGGCAAGCTGACCCTGAA

GTTCATCTGCACCACCGGCAAGCTGCCCGTGCCCTGGCCCACCCTCGTGA

CCACCCTGACCTACGGCGTGCAGTGCTTCAGCCGCTACCCCGACCACATG

AAGCAGCACGACTTCTTCAAGTCCGCCATGCCCGAAGGCTACGTCCAGGA

GCGCACCATCTTCTTCAAGGACGACGGCAACTACAAGACCCGCGCCGAGG

TGAAGTTCGAGGGCGACACCCTGGTGAACCGCATCGAGCTGAAGGGCATC

GACTTCAAGGAGGACGGCAACATCCTGGGGCACAAGCTGGAGTACAACTA

CAACAGCCACAACGTCTATATCATGGCCGACAAGCAGAAGAACGGCATCA

AGGTGAACTTCAAGATCCGCCACAACATCGAGGACGGCAGCGTGCAGCTC

GCCGACCACTACCAGCAGAACACCCCCATCGGCGACGGCCCCGTGCTGCT

GCCCGACAACCACTACCTGAGCACCCAGTCCGCCCTGAGCAAAGACCCCA

ACGAGAAGCGCGGTCACATGGTCCTGCTGGAGTTCGTGACCGCCGCCGGG

ATCACTCTCGGCATGGACGAGCTGTACAAGTAAAGCGGCCGCGACTCTAG

ATCATAATCAGC

**38 GFP variants**

>E1

GGATCCACCGGTCGCCACCATGGTGAGCAAGGGCGAGGAGCTGTTCACTG

GGGTGGTGCCCATCCTGGTCGACGGCGACATAAACGGCCACAAGTTCAGC

GTGTCCGACGAGGGCGGGGGCGATGCCACCTACGGCAAGCTGACCCTGAA

GTTCATCTGCACCACCGGCAAGCTGCCCGTGCCCTGGCCCACCCTCGTGA

CCACCCTGACCTACGGCGTGCAGTGCTTCAGCCGCTACCCCGACCACATG

AAGCAGCACGACTTCTTCAAGTCCGCCATGCCCGAAGGCTACGTCCAGGA

GCGCACCATCTTCTTCAAGGACGACGGCAACTACAAGACCCGCGCCGAGG

TGAAGTTCGAGGGCGACACCCTGGTGAACCGCATCGAGCTGAAGGGCATC

GACTTCAAGGAGGACGGCAACATCCTGGGGCACAAGCTGGAGTACAACTA

CAACAGCCACAACGTCTATATCATGGCCGACAAGCAGAAGAACGGCATCA

AGGTGAACTTCAAGATCCGCCACAACATCGAGGACGGCAGCGTGCAGCTC

GCCGACCATTACCAGCAGAACACCCCCATCGGCGACGGCCCCGTGCTGCT

GCCCGACAACCACTACCTGAGCACCCAGTCCGCCCTGAGCAAAGACCCCA

ACGAGAAGCGCGGTCACATGGTCCTGCTGGAGTTCGTGACCGTCGCCGGG

ATCACTCTCGGCATGGACGAGCTGTACAAGTAAAGCGGCCGCGACTCTAG

ATCATAATCAGC

>E2

GGATCCACCGGTCGCCACCATGGTGAGCAAGGGCGAGGAGCTGTTCACTG

GGGTGGTGCCCATCCTGGTCGACGGCGACATAAACGGCCACAAGTTCAGC

GTGTCCGGCGAGGGCGGGGGCGATGCCACCTACGGCAAGCTGACCCTGAA

GTTCATCTGCACCACCGGCAAGCTGCCCGTGCCCTGGCCCACCCTCGTGA

CCACCCTGACCTACGGCGTGCAGTGCTTCAGCCGCTACCCCGACCACATG

AAGCAGCACGACTTCTTCAAGTCCGCCATGCCCGAAGGCTACGTCCAGGA

GCGCACCATCTTCTTCAAGGACGACGGCAACTACAAGACCCGCGCCGAGG

TGAAGTTCGAGGGCGACACCCTGGTGAACCGCATCGAGCTGAAGGGCATC

GACTTCAAGGAGGACGGCAACATCCTGGGGCACAAGCTGGAGTACAACTA

CAACAGCCACAACGTCTATATCATGGCCGACAAGCAGAAGAACGGCATCA

AGGTGAACTTCAAGATCCGCCACAACAACGAGGACGGCAGCGTGCAGCTC

GCCGACCACTACCAGCAGAACGCCCCCATCGGCGACGGCCCCGTGCTGCT

GCCCGACAACCACTACCTGAGCACCCAGTCCGCCCTGAGCAAAGACCCCA

ACGAGAAGCGCGGTCACATGGTCCTGCTGGAGTTCGTGACCGCCGCCGGG

ATCACTCTCGGCATGGACGAGCTGTACAAGTAAAGCGGCCGCGACTCTAG

ATCATAATCAGC

>E3

GGATCCACCGGTCGCCACCATGGTGAGCAAGGGCGAGGAGCTGTTCACTG

GGGTGGTGCCCATCCTGGTCGACGGCGACATAAACGGCCACAAGTTCAGC

GTGTCCGGCGAGGGCGGAGGCGATGCCACCTACGGCAAGCTGACCCTGAA

GTTCATCTGCACCACCGGCAAGCTGCCCGTGCCCTGGCCCACCCTCGTGA

CCACCCTGACCTACGGCGTGCAGTGCTTCAGCCGCTACCCCGACCACATG

AAGCAGCACGACTTCTTCAAGTCCGCCATGCCCGATGGCTACGTCCAGGA

GCGCACCATCTTCTTCAAGGACGACGGCAACTACAAGACCCGCGCCGAGG

TGAAGTTCGAGGGCGACACCCTGGTGAACCGCACCGAGCTGAAGGGCATC

GACTTCAAGGAGGACGGCAACATCCTGGGGCACAAGCTGGAGTACAACTA

CAACAGCCACAACGTCTATATAATGGCCGACAAGCAGAAGAACGGCATCA

AGGTGAACTTCAAGATCCGCCACAACATCGAGGACGGCAGCGAGCAGCTC

GCCGTCCACTACCAGCAGAACACCCCCATCGGCGACGGCCCCGTGCTGCT

GCCCGACAACCACTACCTGAGCACCCAGTCCGCCCTGAGCAAAGACCCCA

ACGAGAAGCGCGGTCACATGTCCTGCTGGAGTTCGTGACCGCCGCCGGGA

TCACTCTCGGCATGGACGAGCTGTACAAGTAAAGCGGCCGCGACTCTAGA

TCATAATCAGC

>E4

GGATCCACCGGTCGCCACCATGGTGAGCAAGGGCGAGGAGCTGTTCACTG

GGGTGGTGCCCATCCTGGTCGACGGCGACATAAACGGCCACAAGTTCAGC

GTGTCCGGCGAGGGCGGGGGCGATGCCACCTACGGCAAGCTGACCCTGAA

GTTCATCTGCACCACCGGCAAGCTGCCCGTGCCCTGGCCCACCCTCGTGA

CCACCCTGACCTACGGCGTGCAGTGCTTCAGCCGCTACCCCGACCACATG

AAGCAGCACGACTTCTTCAAGTCCGCCATGCCCGAAGGCTACGTCCAGGA

GCGCACCATCTTCTTCAAGGACGACGGCAACTACAAGACCCGCGCCGAGG

TGAAGTTCGAGGGCGACACCCTGGTGAACCGCATCGAGCTGAAGGGCATC

GACTTCAAGGAGGACGGCAACATCCTGGGGCACAAGCTGGAGTACAACTA

CAACAGCCACAACGTCTATATCATGGCCGACAAGCAGAAGAACGGCATCA

AGGTGAACTTCAAGATCCGCCACAACATCGAGGACGGCAGCGTGCAGCTC

GCCGACCACTACCAGCAGAACACCCCCATCGGCGACGGCCCCGTGCTGCT

GCCCGACAACCACTACCTGAGCACCCAGTCCGCCCTGAGCAAAGACCCCA

ACGAGAAGCGCGGTCACATGGTCCTGCTGGAGTTCGTGACCGCCGCCGGG

AACACTCTCGGCATGGACGAGCTGTACAAGTAAAGCGGCCGCGACTCTAG

ATCATAATCAGC

>E5

GGATCCACCGGTCGCCACCATGGTGAGCAAGGGCGAGGAGCTGTTCACTG

GGGTGGTGCCCATCCTGGTCGACGGCGACATAAACGGCCACAAGTTCAGC

GTGTCCGGCGAGGGCGGGGGCGATGCCACCTACGGCAAGCTGACCCTGAA

GTTCATCTGCACCACCGGCAAGCTGCCCGTGCCCTGGCCCACCCTCGTGA

CCACCCTGACCTACGGCGTGCAGTGCTTCAGCCGCTACCCCGACCACATG

AAGCAGCACGACTTCTTCAAGTCCGCCATGCCCGAAGGCTACGTCCAGGA

GCGCACCATCTTCTTCGAGGACGACGGCAACTACAAGACCCGCGCCGAGG

TGAAGTTCGAGGGCGACACCCTGGTGAACCGCATCGAGCTGAAGGGCATC

GACTTCAAGGAGGACGGCAACATCCTGGGGCACAAGCTGGAGTACAACTA

CAACAGCCACAACGTCTATATCATGGCCGACAAGCAGAAGAACGGCATCA

AGGTGAACTTCAAGATCCGCCACAACATCGATGACGGCAGCGTGCAGCTC

GCCGACCACTACCAGCAGAACACCCCCATCGGCGACGGCCCCGTGCTGCT

GCCCGACAACCACTACCTGAGCACCCAGTCCGCCCTGAGCAAAGACCCCA

ACGAGAAGCGCGGTCACATGGTCCTGCTGGAGTTCGTGACCGCCGCCGGG

ATCACTCTCGGCATGGACGAGCTGTACAAGTAAAGCGGCCGCGACTCTAG

ATCATAATCAGC

>E6

GGATCCACCGGTCGCCACCATGGTGAGCAAGGGCGAGGAGCTGTTCACTG

GGGTGGTGCCCATCCTGGTCGACGGCGACATAAACGGCCACAAGTTCAGC

GTGTCCGGCGAGGGCGGGGGCGATGCCACCTACGGCAAGCTGACCCTGAA

GTTCATCTGCACCACCGGCAAGCTGCCCGTGCCCTGGCCCACCCTCGTGA

CCACCCTGACCTACGGTGTGCAGTGCTTCAGCCGCTACCCCGACCACATG

AAGCAGCACGACTTCTTCAAGTCCGCCATGCCCGAAGGCTACGTCCAGGA

GCGCACCATCTTCTTCAAGGACGACGGCAACTACAAGACCCGCGCCGAGG

TGAAGTTCGAGGGCGACACCCTGGTGAACCGCATCGAGCTGAAGGGCATC

GACTTCAAGGAGGACGGCAACATCCTGGGGCACAAGCTGGAGTACAACTA

CAACAGCCACAACGTCTATATCATGGCCGACAAGCAGAAGAACGGCATCA

AGGTGAACTTCAAGATCCGCCACAACATCGAGGACGGCAGCGTACAGCTC

GCCGACCACTACCAGTAGAACACCCCCATCGGCGACGGCCCCGTGCTGCT

GCCCGACAACCACTACCTGAGCACCCAGTCCGCCCTGAGCAAAGACCCCA

ACGAGAAGCGCGGTCACATGGTCCTGCTGGAGTTCGTGACCGCCGCCGGG

ATCACTCTCGGCATGGACGAGCTGTACAAGTAAAGCGGCCGCGACTCTAG

ATCATAATCAGC

>E7

GGATCCACCGGTCGCCACCATGGTGAGCAAGGGCGAGGAGCTGTTCACTG

GGGTGGTGCCCATCCTGGTCGACGGCGACATAAACGGCCACAAGTTCAGC

GTGTCCGGCGAGGGCGGGGGCGATGCCACCTACGGCAAGCTGACCCTGAT

GTTCATCTGCACCACCGGCAAGCTGCCCGTGCCCTGGCCCACCCTCGTGA

CCACCCTGACCTACGGCGTGCAGTGCTTCAGCCGCTACCCCGACCACATG

AAGCAGCACGACTTCTTCAAGTCCGCCATGCCCGAAGGCTACGTCCAGGA

GCGCACCATCTTCTTCAAGGACGACGGCAACTACAAGACCCGCGCCGAGG

TGAAGTTCGAGGGCGACACCCTGGTGAACCGCATCGAGCTGAAGGGCATC

GACTTCAAGGAGGACGGCAACATCCTGGGGCACAAGCTGGAGTACAACTA

CAACAGCCACAACGTCTATATCATGGCCGACAAGCAGAAGAACGGCATCA

AGGTGAACTTCAAGATCCGCCACAACATCGAGGACGGCAGCGTGCAGCTC

GCCGACCACTACCAGCAGAACACCCCCATCGGCGACGGCCCCGTGCTGCT

GCCCGACAACCACTACCTGAGCACCCAGTCCGCCCTGAGCAAAGACCCCA

ACGAGAAGCGCGGTCACATGGTCCTGCTGGAGTTCGTGACCGCCGCCGGG

ATCACTCTCGGCATGGACGAGCTGTACAAGTAAAGCGGCCGCGACTCTAG

ATCATAATCAGC

>E8

GGATCCACCGGTCGCCACCATGGTGAGCAAGGGCGAGGAGCTGTTCACTG

GGGTGGTGCCCATCCTGGTCGACGGCGACATAAACGGCCACAAGTTCAGC

GTGTCCGGCGAGGGCGGGGGCGATGCCACCTACGGCAAGCTGACACTGAA

GTTCATCTGCACCACCGGCAAGCTGCCCGTGCCCTGGCCCACCCTCGTGA

CCACCCTGACCTACGGCGTGCAGTGCTTCAGCCGCTACCCCGACCACATG

AAGCAGCACGACTTCTTCAAGTCCGCCATGCCCGAAGGCTACGTCCAGGA

GCGCACCATCTTCTTCAAGGACGACGGCAACTACAAGACCCGCGCCGAGG

TGAAGTTCGAGGGCGACACCCTGGTGAACCGCATCGAGCTGAAGGGCATC

GACTTCAAGGAGGACGGCAACATCCTGGGGCACAAGCTGGAGTACAACTA

CAACAGCCACAACGTCTATATCATGGCCGACAAGCAGAGGAACGGCATCA

AGGTGAACTTCAAGATCCGCCACAACATCGAGGACGGCAGCGTGCAGCTC

GCCGACCACTACCAGCAAAACACCCCCATCGGCGACGGCCCCGTGCTGCT

GCCCGACAACCACTACCTGAGCACCCAGTCCGCCCCTGAGCAAAGACCCC

AACGAGAAGCGCGGTCACATGGTCCTGCTGGAGTTCGTGACCGCCGCCGG

GATCACTCTCGGCATGGACGAGCTGTACAAGTAAAGCGGCCGCGACTCTA

GATCATAATCAGC

>E9

GGATCCACCGGTCGCCACCATGGTGAGCAAGGGCGAGGAGCTGTTCACTG

GGGTGGTGCCCATCCTGGTCGACGGCGACATAAACGGCCACGAGTTCAGC

GTGTCCGGCGAGGGCGGGGGCGATGCCACCTACGGCAAGCTGACCCTGAA

GTTCATATGCACCACCGGCAAGCTGCCCGTGCCCTGGCCCACCCTCGTGA

CCACCCTGACCTACGGCGTGCAGTGCTTCAGCCGCTACCCCGACCACTTG

AAGCAGCACGACTTCTTCAAGTCCGCCATGCCCGAAGGCTACGTCCAGGA

GCGCACCATCTTCTTCAAGGACGACGGCAACTACAAGACCCGCGCCGAGG

TGAAGTTCGAGGGCGACACCCTGGTGAACCGCATCGAGCTGAAGGGCATC

GACTTCAAGGAGGACGGCAACATCCTGGGGCACAAGCTGGAGTACAACTA

CATCAGCCACAACATCTATATCATGGCCGACAAGCAGAAGAACGGCATCA

AGGTGAACTTCAAGATCCGCCACAACATCGAGGACGGCAGCGTGCAGCTC

GCCGACCACTACCAGCAGAACACCCCCATCGGCGACGGCCCCGTGCTGCT

GCCCGACAACCACTACCTGAGCACCCAGTCCGCCCTGAGCAAAGACCCCA

ACGAGAAGCGCGGTCACATGGTCCTGCTGGAGTTCGTGACCGCCGCCGGG

ATCACTCTCGGCATGGACGAGCTGTACAAGTAAAGCGGCCGCGACTCTAG

ATCATAATCAGC

>E10

GGATCCACCGGTCGCCACCATGGTGAGCAAGGGCGAGGAGCTGTTCACTG

GGGTGGTGCCCATCCTGGTCGACGGCGACATAAACGGCCACAAGTTCAGC

GTGTCCGGCGAGGGCGGGGGCGATGCCACCTACGGCAAGCTGACCCTGAA

GTTCATCTGCACCACCGGCAAGCTGCCCGTGCCCTGGCCCACCCTCGTGA

CCACCCTGACCTACGGCGTGCAGTGCTTCAGCCGCTACCCCGACCACATG

AAGCAGCACGACTTCTTCAAGTCCGCCATGCCCGAAGGATACGTCCAGGA

GCACACCATCTTCTTCAAGGACGACGGCAACTACAAGACCCGCGCCGAGG

TGAAGTTCGAGGGCGACACCCTGGTGAACCGCATCGAGCTGAAGGGCATC

GACTTCAAGGAGGACGGCAACATCCTGGGGCACAAGCTGGAGTACAACTA

CAACAGCCACAACGTCTATATCATGGCCGACAAGCAGAAGAACGGCATCA

AGGTGAACTTCAAGATCCGCCACAACATCGAGGACGGCAGCGTGCAGCTC

GCCGACCACTACCAGCAGAACACCCCCATCGGCGACGGCCCCGTGCTGCT

GCCCGACAACCACTACCTGAGCACCCAGTCCGCCCTGAGCAAAGACCCCA

ACGAGATGCGCGGTCACATGGTCCTGCTGGAGTTCGTGACCGCCGCCGGG

ATCACTCTCGGCATGGACGAGCTGTACAAGTAAAGCGGCCGCGACTCTAG

ATCATAATCAGC

>E13

GGATCCACCGGTCGCCACCATGGTGAGCAAGGGCGAGGAGCTGTTCACTG

GGGTGGTGCCCATCCTGGTCGACGGCGACATAAACGGCCACAAGTTCAGC

GTGTCCGGCGAGGGCGGGGGCGATGCCACCTACGGCAAGCTGACCCTGAA

GTTCATCTGCACCACCGGCAAGCTGCCCGTGCCCTGGCCCACCCTCGTGA

CCACCCTGACCTACGGCGTGCAGTGCTTCAGCCGCTACCCCGACCACATG

AAGCAGCACGACTTCTTCAAGTCCGCCATGCCCGAAGGCTACGTCCAGGA

GCGCACCATCTTCTTCAAGGACGACGGCAACTACAAGACCCGCGCCGAGG

TGAAGTTCGAGGGCGACACCCTGGTGAACCGCATCGAGCTGAAGGGCATC

GACTTCAAGGAGGACGGCAAAATCCTGGGGCACAAGCTGGAGTACAACTA

CAACAGCCATAACGTCTATATCATGGCCGACAAGCAGAAGAACGGCATCA

AGGTGAACTTCAAGATCCGCCACAACATCGAGGACGGCAGCGTGCAGCTC

GCCGACCACTACCAGCAGAACACCCCCATCGGCGACGGCCCCGTGCTGCT

GCCCGACAACCACTACCTGAGCCCCCAGTCCGCCCTGAGCAAAGACCCCA

ACGAGAAGCGCGGTCACATGGTCCTGCTGGAGTTCGTGACCGCCGCCGGG

ATCACTCTCGGCATGGACGAGCTGTACAAGTAAAGCGGCCGCGACTCTAG

ATCATAATCAGC

>E14

GGATCCACCGGTCGCCACCATGGTGAGCAAGGGCGAGGAGCTGTTCACTG

GGGTGGTGCCCATCCTGGTCGACGGCGACATAAACGGCCACAAGTTCAGC

GTGTCCGGCGAGGGCGGGGGCGATGCCACCTACGGCAAGCTGACCATGAA

GTTCATCTGCACCACCGGCAAGCTGCCCGTGCCCTGGCCCACCCTCGTGA

CCACCCTGACCTACGGCGTGCAGTGCTTCAGCCGCTACCCCGACCACATG

AAGCAGCACGACTTCTTCAAGTCCGCCATGCCCGAAGGCTACGTCCAGGA

GCGCACCATCTTCTTCAAGGACGACGGCAACTACAAGACCCGCGCCGAGG

TGAAGTTCGAGGGCGACACCCTGGTGAACCGCATCGAGCTGAAGGGCATC

GACTTCAAGGAGGACGGCAACATCCTGGGGCACAAGCTGGAGTACAACTA

CAATAGCCACAACGTCTATATCATGGCAGACAAGCGGAAGAACGGCATCA

AGGTGAACTTCAAGATCCGCCACAACATCGAGGACGGCAGCGTGCAGCTC

GCCGACCACTACCAGCAGAACACCCCTATCGGCGACGGCCCCGTGCTGCT

GCCCGACAACCACTACCTGAGCACCCAGTCCGCCCTGAGCAAAGACCCCA

ATGAGAAGCGCGGTCACATGGTCCTGCTGGAGTTCGTGACCGCCGCCGGG

ATCACTCTCGGCATGGACGAGCTGTACTAGTAAAGCGGCCGCGACTCTAG

ATCATAATCAGC

>E15

GGATCCACCGGTCGCAACCATGGTGAGCAAGAGCGAGGTGCTGTTCACTG

GGGTGGTGCTCACCCTGGTCGACGGCGACATAAACGGCCACAAGTTCAGC

GTGTCCGGCGAGGGCGGGGGCGATGCCACCTACGGCAAGCTGACCCTGAA

GTTCATCTGCACCACCGGCAAGCTGCCCGTGCCCTGGCCCACCCTCGTGA

CCACCCTGACCTACGGCGTACAGTGCTTCAGCCGCTACCCCTACCACATG

AAGCAGCACGACTTCTTCAAGTCCGCCATGTCCGAAGGCTACGTCCAGGA

GCGCACCATCTTCTTCAAGGACGACGGCAACTACAAGACCCGCGCCGAGG

TGAAGTTCGAGGGCGACACCCTGGTGAACCGCATCGAGCTGAAGGGCATC

GACTTCAAGGAGGACGGCAACATCCTGGGGCACAAGCTGGAGTACAACTA

CAACAACCACAACGTCTATGTCATGGCCGACAAGCAGAAGAACGGCATCA

AGGTGAACTTCAAGATCCGCCACAACATCGAGGACGGCAGCGTGCAGCTC

GCCGACCACTACCAGCAGAACACCCCCATCGGCGACGGCCCCGTGCTGCT

GCCCGACAACCACTACCTGAGCACCCAGTCCGCCCTGAGCAAAGACCCCA

ACGAGAAGCGCGGTCACATGGTCCTGCTGGAGTTCGTGACCGCCGCCGGG

ATCACTCTCGGCATGGACGAGCTGTACAAGTAAAGCGGCCGCGACTCTAG

ATCATAATCAGC

>E16

GGATCCACCGGTCGCCACCATGGTGAGCAAGGGCGAGGAGCTGTTCACTG

GGGTGGTGCCCATCCTGGTCGACGGCGACATAAACGGCCACAAGTTCAGC

GTGTCCGGCGAGGGCGGGGGCGATGCCACCTACGGCAAGCTGACCCTGAA

GTTCATCTGCACCACCGGCAAGCTGCCCGTGCCCTGGCCCACCCTCGTGA

CCACCCTGACCTACGGCGTGCAGTGCTTCAGCCGCTACCCCGACCACATG

AAGCAGCACGACTTCTTCAAGTCCGCCATGCCCGAAGGCTACGTCCAGGA

GCGCACCATCTTCTTCAAGGACGACGGCAACTACAAGACCCGCGCCTAGG

TGAAGTTCGAGGGCGACACCCTGGTGAACCGCATCGAGCTGAAGGGCATC

GACTTCAAGGAGGACGGCAACATCCTGGGGCACAAGCTGGAGTACAACTA

CAACAGCCACAACGTCTATATCATGGCCGACAAGCAGAAGAACGGCATCA

AGGTGTACTTCAAGATCCGCCACAACATCGAGGACGGCAGCGTGCAGCTC

GCCGACCACTACCAGCAGAACACCCCCATCGGCGACGGCCCCGTGCTGCT

GCCCGACAACCACTACCTGAGCACCCAGTCCGCCCTGAGCAAAGACCCCA

ACGAGAAGCGCGGTCACATGGTCCTGCTGGAGTTCGTGGCCGCCGCCGGG

ATGACTCTCGGCATGGACGAGCTGTACAAGTAAAGCGGCCGCGACTCTAG

ATCATAATCAGC

>E17

GGATCCACCGGTCGCCACCATGGTAAGCAAGGGCGAGGAGCTGTTCACTG

GGGTGGTGCCCATCCTGGTCGACGGCGACATAAATGGCACAAGTTCAGCG

TGTCCGGCGAGGGCGGGGGCGATGCCACCTACGGCAAGCTGACCCTGAAG

TTCATCTGCACCACCGGCAAGCTGCCCGTGCTCTGGCCCACCCTCGTGAC

CACCCTGACCTACGGCGTGCAGTGCTTCAGCCGCTACCCCGACCACATGA

AGCAGCACGACTTCTTCAAGTCCGCCATGCCCGAAGGCTACATCCAGGAG

CGCACCATCTTCTTCAAGGACGACGGCAACTACAAGACCCGCGCCGAGGT

GATGTTCGAGGGCGACACCCTGGTGAACCGCATCGAGCTGAAGGGCATCG

ACTTCAAGGAGGACGGCAACATCCTGGGGCACAAGCTGGAGTACAACTAC

AACAGCCACAACGTCTATATCATGGCCGACAAGCAGAAGAACGGCATCAA

GGTGAACTTCAAGATCCGCCACAACATCGAGGACGGCAGCGTGCAGCTCG

CCGACCACTACCAGCAGAACACCCCCATCGGCGACGGCCCCGTGCTGCTG

CCCGACAACCACTACCTGAGCACCCAGTCCGCCCTGAGCAAAGACCCCAA

CGAGAAGCGCGGTCACATGGTCCTGCTGGAGATCGTGACCGCCGCCGGGA

TCACTCTCGGCATGGACGAGCTGTACAAGTAAAGCGGCCGCGACTCTAGA

TCATAAACAGC

>E18

GGATCCACCGGTCGCCACCATGGTGAGCAAGGGCGAGGAGCTGTTCACTG

GGGTGGTGCCCATCCTGGTCGTCGGCGACATAAACGGCCACAAGTTCAGC

GTGTCCGGCGAGGGCGGGGGCGATGCCACCTACGGCAAGCTGACCCTGAA

GTTCATCTGCACCACCGGCAAGCTGCCCGTGCCCTGGCCCACCCTCGTGA

CCACCCTGACCTACGGCGTGCAGTGCTTCAGCCGCTACCCCGGCCACATG

AAGCAGCACGACATCTTCAAGTCCGCCATGCCCGAAGGCTACGTCCAGGA

GCGCACCATCTTCTTCAAGGACGACGGCAACTACAAGACCCGCGCCGAGG

TGAAGTTCGAGGGCGACACCCCGGTGAACCGCATCGAGCTGAAGGGCATC

GACTTCATGGAGGACGGCAACATCCTGGGGCACAAGCCGGAGTATAACTA

CAACAGCCACAACGTCTATATCATGGCCGACAAGCAGAAGAACGGCATCA

AGGTGAACTTCAAGATCCGCCACAACATCGAGGACGGCAGCGTGCAGCTC

GCCGACCACTACCAGCAGAACACCCCCATCGGCGACGGCCCCGTGCTGCT

GCCCGACAACCACTACCTGAGCACCCAGTCCGCCCTGAGCAAAGACCCCA

ACGAGAAGCGCGGTCACATGGTCCTGCTGGAGTTCGTGACCGCCGCCGGG

ATCACTCTCGGCATGGACGAGCTGTACAAGTAAAGCGGCCGCGACTCTAG

ATCATAATCAGC

>E19

GGATCCACCGGTCGCCACCATGGTGAGCAAGGGCGAGGAGCTGTTCACTG

GGGTGGTGCCCATCCTGGTCGACGGCGACATAAACGGCCACAAGTTCAGC

GTGTCCGGCGAGGGCGGGGGCGATGCCACCTACGGCAAGCTGACCCTGAA

GTTCATCTGCACCACCGGCAAGCTGCCCGTGCCCTGGCCCACCCTCGTGG

CCACCCTGACCTACGGCGTGCAGTGCTTCAGCCGCTACCCCGACCACATG

AAGCAGCACGACTTCTACAAGTCCGCCATGCCCGAAGGCTACGTCCAGGA

GCGCACCATCTTCTTCAAGGACGACGGCAACTACAAGACCCGCGCCGAGG

TGAAGTTCGAGGGCGACACCCTGGTGAACCGCATCGAGCTGAAGGGCATC

GACTTCAAGGAGGACGTCAACATCCTGGGGCACAAGCTGGAGTACAACTA

CAACAGCCACAACGTCTATATCATGGCCGACAAGCAGAAGAACGGCATCA

AGGTGAACTTCAAGATCCGCCACAACATCGAGGACGGCAGCGTGCAGCTC

GCCGACCACTACCAGCAGAACACCCCCATCGGCGACGGCCCCGTGCTGCT

GCCCGACAACCACTACCTGAGCACCCAGTCCGCCCTGAGCAAAGACCCCA

ACGAGAAGCGCGGTCACATGGTCCTGCTGGAGTTCGTGACCGCCGCCGGG

ATCACTCTCGGCATGGACGAGCTGTACAAGTAAAGCGGCCGCGACTCTAG

ATCATAATCAGC

>E21

GGATCCACCGGTCGCCACCATGGTGAGCAAGGGCGAGGAGCTGTTCACTG

GGGTGGTGCCCATCCTGGTCGACGGCGACATAAACGGCCACAAGTTCAGC

GTGTCCGGCGAGGGCGGGGGCGATGCCACCTACGGCAAGCTGACCCTGAA

GTTCATCTGCACCACCGGCAAGCTGCCCGTGCCCTGGCCCACCCTCGTGA

CCACCCTGACCTACGGCGTGCAGTGCTTCAGCCGCTACCCCGACCACATG

AAGCAGCACGACTTCTTCAAGTCCGCCATGCCCGAAGGCTACGTCCAGGA

GCGTACCATCTTCTTTAAGGACGACGGCAACTACAAGACCCGCGCCGAGG

TGAAGTTCGAGGGCGACACCCTGGTGAACCGCATCGAGCTGAAGGGCATC

GACTTCAAGGAGGACTGCAACATCCTGGGGCACAAGCTGGAGTACAACTA

CAACAGCCACAACGTCTATATCATGGCCGACAAGCAGAAGAACGGCATCA

AGGTGAACTTCAAGATCCGCCACAACATCGAGGACGGCAGCGTGCAGCTC

GCCGACCACTACCAGCAGAACACCCCCATCGGCGACGGCCCCGTGCTGCT

GCCCGACAACCACTACCTGAGCACCCAGTCCGCCCCGAGCAAAGACCCCA

ACGAGAAGCGCGGTCACATGGTCCTGCTGGAGTTCGTGACCGCCGCCGGG

ATCACTCTCGGCATGGACGAGCTGTACAAGTAAAGCGGCCGCGACTCTAT

ATCATAATCAGC

>E22

GGATCCACCGGTCGCCACCATGGTGAGCTAGGGCGAGGAGCTGTTCACTG

GGGTGGTGCCCATCCTGGTCGACGGCGACATAAACGGCCACAAGTTCAGC

GTGTCCGGCGAGGGCGGGGGCGATGCCACCTACGGCAAGCTGACCCTGAA

GTTCATCTGCACCACCAGCAAGCTGCCCGTGCCCTGGCCCACCCTCGTGA

CCACCCTGACCTACGGCGTGCAGTGCTTCAGCCGCTACCCCGACCACATG

AAGCAGCACGACTTCTTCAAGTCCGCCATGCCCGAAGGCTACGTCCAGGA

GCGCACCATCTTCTTCAAGGACGACGGCAACTACAAGACCCGCGCCGAGG

TGAAGTTCGAGGGCGACACCCTGGTGAACCGCATCGAGCTGAAGGGCATC

GACTTCAAGGAGGACGGCAACATCCTGGGGCACAAGCTGGAGTACAACTA

CAACAGCCACAACGTCTATATCATGGCCGACAAGCAGAAGAACGGCATCA

AGGTGAACTTCAAGATCCGCCACAACATCAAGGACGGCAGCGTGCAGCTC

GCCGACCACTACCAGCAGAACACCCCCATCGGCGACGGCCCCGTGCTGCT

GCCCGACAACCACTACCTGAGCACCCAGTCCGCCCTGAGCAAAGACCCCA

ACAAGAAGCGCGGTCACATGGTCCTGCTGGAGTTCGTGACCGCCGCCGGG

ATCACTCTCGGCATGGACGAGCTGTACTAGTAAAGCGGCCGCGACTCTAG

ATCATAATCAGC

>E23

GGATCCACCGGTCGCCACCATGGTGAGCAAGGGCGAGGAGCTGTTCACTG

GGGTGGTGCCCATCCTGGTCGACGGCGACATAAACGGCCACAAGTTCAGC

GTGTCCGGCGAGGGCGGGGGCGATGACACCTACGGCAAGCTGACCCTGAA

GTTCATCTGCACCACCGGCAAGCTGCCCGTGCCCTGGCCCACCCTCGTGA

CCACCCTGACCTACGGCGTGCAGTGCTTCAGCCGCTACCCCGACCACATG

AAGCAGCACGACTTCTTCAAGTCCGCCATGCCCGAAGGCTACGTCCAGGA

GCGCACCATCTTCTTCAAGGACGACGGCAACTACAAGACCCGCGCCGAGG

TGAAGTTCGAGGGCGACACCCTGGTGAACCGCATCGAGCTGAAGGGCATC

GACTTCAAGGAGGACGGCAACATCCTGGGGCACAAGCTGGAGTACAACTT

CAACAGCCACAACGTCTATATCATGGCCGACAAGCAGAAGAACGGCATCA

AGGTGAACTTCAAGATCCGCCACAACATCGAGGACGGCAGCGTGCAGCTC

GCCGACCACTACCAGCAGAACACCCCCATCGGCGACGGCCCCGTGCTGCT

GCCCGACAACCACTACCTGAGCACCCAGTCCGCCCTGAGCAAAGACCCCA

ACGAGAAGCGCGGTCACATGGTCCTGCTGGAGTTCGTGACCGCCGCCGGG

ATCACTCTCGGCATGGACGAGTTGTACAAGTAAAGCGGCCGCGACTCTAG

ATCATAATCAGC

>E24

GGATCCACCGGTCGCCACCATGGTGAGCAAGGGCGAGGAGCTGTTCACTG

GGGTGGTGCCCATCCTGGTCGACGGCGACATAAACGGCCACAAGTTCAGC

GTGTCCGGCGAGGGCGGGGGCGATGCCACCTACGGCAAGCTGACCCTGAA

GTTCATCTGCACCACCGGCAAGCTGCCCGTGCCCTGGCCCACCCTCGTGA

CCACCCTGACCTACGGCGTGCAGTGCTTCAGCCGCTACCCCGACCACATG

AAGCAGCACGACTTCTTCAAGTCCGCCATGCCCGAAGGCTACGTCCAGGA

GCGCACCATCTTCTTCAAGGACGACGGCAACTACAAGACCCGCGCCGAGG

TGAAGTTCGAGGGCGACACCCTGGTGAACCGCATCGAGCTGAAGGGCATC

GACTTCAAGGAGGACGGCAACATCCTGGGGTACAAGCTGGAGTACAACTA

CAACAGCCACAACGTCTATATCATGGCCGACAAGCAGAAGAACGGCATCA

AGGTGAACTTCAAGATCCACCACAACATCGAGGACGGCAGCGTGCAGCCC

GCCGACCACTACCAGCAGAACACCCCCATCGGCGACGGCCCCGTGCTGCT

GCCCGACAACCACTACCTGAGCACCCAGTCCGCCCTGAGCAAAGACCCCA

ACGAGAAGCGCGGTCACATGGTCCTGCTGGAGTTCGTGACCGCCGCCGGG

ATCACTCTCGGCATGGACGAGCTGTACAAGTAAAGCGGCCGCGACTCTAG

ATCATAATCAGC

>E25

GGATCCACCGGTCGCCACCATGGTGAGCAAGGGCGAGGAGCTGTTCACTG

GGGTGGTGCCCATCCTGGTCGACGGCGACATAAACGGCCACAAGTTCAGC

GTGTCCGGCGAGGGCGGGGGCGATGCCACCTACGGCAAGCTGACCCTGAA

GTTCATCTGCACCACCGGCAAGCTGCCCGTGCCCTGGCCCACCCTCGTGA

CCACCCTGACCTACGGCGTGCAGTGCTTCAGCCGCTACCCCGACCACATG

AAGCAGCACGACTTCTTCAAGTCCGCCATGCCCGAAGGCTACGTCCAGGA

GCGCTCCATCTTCTTCAAGGACGACGGCAACTACAAGAGCCGCGCCGAGG

TGAAGTTCGAGGGCGACACCCTGGTGAACCGCATCGAGCTGAAGGGCATC

GACTTCTAGGAGGACGGCAACATCCTGGGGCACAAGCTGGAGTACAACTA

CAACAGCCACAACGTCTATATCATGGCCGACAAGCAGAAGAACGGCATCA

AGGTGAACTTCAAGATCCGCCACAACATCGAGGACGGCAGCGTGCAGCTC

GCCGACCACTACCAGCAGAACACCCCCATCGGCGACGGCCCCGTGCTGCT

GCCCGACAACCACTACCCGAGCACCCAGTCCGCCCTGAGCAAAGACCCCA

ACGAGAAGCGCGGTCACATGGTCCTGCTGGAGTTCGTGACCGCCGCCGGG

ATCACTCTCGGCATGGACGAGCTGTACAAGTAAAGCGGCCGCGACTCTAG

ATCATAATCAGC

>E26

GGATCCACCGGTCGCCACCATGGTGAGCAAGGGCGAGGAGCTGTTCACTG

GGGTGGTGCCCATCCTGGTCGACGGCGACATAAACGGCCACAAGTTCAGC

GTGTCCGGCGAGGGCGGGGGCGATGCCACCTACGGCAAGCTGACCCTGAA

GTTCATTTGCACCACCGGCGAGCTGCCCGTGCCCTGGCCCACCCTCGTGA

CCACCCCGACCTACGGCGTGCAGTGCTTCAGCCGCTACCCCGACCACATG

AAGCAGCACGACTTCTTCAAGTCCGCCATGCCCGAAGGCTACGTCCAGGA

GCGCACCATCTTCTTCAAGGACGACGGCAACTACAAGACCCGCGCCGAGG

TGAAGTTCGAGGGCGACACCCTGGTGAACCGCATCGAGCTGAAGGGCATC

GACTTCAAGGAGGACGGCAACATCCTGGGGCACAATCTGGAGTACAACTA

CAACAGCCACAACGTCTATATCATGGCCGACAAGCAGAAGAACGGCATCA

AGGTGAACTTCAAGATCCGCCACAACATCGAGGACGGCAGCGTGCAGCTC

GCCGACCACTACCAGCAGAACACCCCCATCGGCGACGGCCCCGTGCTGCT

GCCCGACAACCACTACCTGAGCACCCAGTCCGCCCTGAGCAAAGACCCCA

ACGAGAAGCGCGGTCACATGGTCCTGCTGGAGTTCGTGACCGCCGCCGGG

ATCACTCTCGGCATGGGCGAGCTGTACAAGTAAAGCGGCCGCGACTCTAG

ATCATAATCAGC

>E27

GGATCCACCGGTCGCCACCATGGTGAGCAAGGGCGAGGAGCTGTTCACTG

GGGTGGTGCCCATCCTGGTCGACGGCGACATAAACGGCCACAAGATCAGC

GTGTCCGGCGAGGGCGGGGGCGATGCCACCTACGGCAAGCTGACCCTGAA

GTTCATCTGCACCACCGGCAAGCTGCCCGTGCCCTGGCCCACCCTCGTGA

CCACCCTGACCTACGGCGTGCAGTGCTTCAGCCGCTACCCCGACCACATG

AAGCAGCACGACTTCTTTTAGTCCGCCATGCCCGAAGGCTACGTCCAGGA

GCGCACCATCTTCTTCAAGGACGACGGCAACTACAAGACCCACGCCGAGG

TGAAGTTCGAGGGCGACACCCTGGTGAACCGCATCGAGCTGAAGGGCATC

GACTTCAAGGAGGACGGCAACATCCTGGGGCACAAGCTGGAGTACAACTA

CAACAGCCACAACGTCTATATCATGGCCGACAAGCAGAAGAACGGCATCA

AGGTGAACTTCAAGATCCGCCACAACATCGAGGACGGCAGCGTGCAGCTC

GCCGACCACTACCAGCAGAACACCCCCATCGGCGACGGCCCCGTGCTGCT

GCCCGACAACCACTACCTGAGCACCCAGTCCGCCCTGAGCAAAGACCCCA

ACGAGAAGCGCGGTCACATGGTCCTGCTGGAGTTCGTGACCGCCGCCGGG

ATCACTCTCGGCATGGACGAGCTGTACAAGTAAAGCGGCCGCGACTCTAG

ATCATAATCAGC

>E28

GGATCCACCGGTCGCCACCATGGTGAGCAAGGGCGAGGAGCTGTTCACTG

GGGTGGTGCCCATCCTGGTCGACGGCGACATAAACGGCCACAAGTTCAGC

GTGTCCGGCGAGGGCGGGGGCGATGCCACCTACGGCAAGCTGACCCTGAA

GTTCATCTGCACCACCGGCAAGCTGCCCGTGTCCTGGCCCACCCTCGTGA

CCACCCTGACCTACGGCGTGCAGTGCTACAGCCGCTACCCCGACCACATG

AAGCAGCACGACTTCTTCAAGTCCGCCATGCCCGAAGGCTACGTCCAGGA

GCGCACCATCTTCTTCAAGGACGACGGCAACTACAAGACCCGCGCCGAGG

TGAAGTTCGAGGGCGACACCCTGGTGAACCGCATCGAGCTGAAGGGCATC

GACTTCAAGGAGGACGGCAACATCCTGGGGCACAAGCTGGAGTACAACTA

CAACAGCCACAACGTCTATATCATGGCCGACAAGCAGAAGAACGGCATCA

AGGTGAACTTCAAGATCCGCCACAACATCGAGGACGGCAGCGTGCAGCTC

GCCGACCACTACCAGCAGAACACCCCCATCGGCGACGGCCCCGTGCTGCT

GCCCGACAACCACTACCTGAGCACCCAGTCCGCCCTGAGCAAAGACCCCA

ACGAGAAGCGCGGTCATATGGTCCTGCTGGAGTTCGTGACCGCCGCCGGG

ATCACTCTCGGCATGGACGAGCTGTACAAGTAAAGCGGCCGCGACTCTAG

ATCATAGTCAGC

>E30

GGATCCACCGGTCGCCACCATGGTGAGCAAGGGCGAGGAGCTGTTCACTG

GGGTGGTGCCCATCCTGGTCGACGGCGACATAAACGGCCACAAGTTCAGC

GTGTCCGGCGAGGGCGGGAGCGATGCCACCTACGGCAAGCTGACCCTGAA

GTTCATCTGCATCACCGGCAAGCTGCCCGTGCCCTGGCCCACCCTCGTGA

CCACCCTGACCTACGGCGTGCAGTGCTTCAGCCGCTACCCCGACCACATG

AAGCAGCACGACTTCTTCAAGTCCGCCATGCCCGAAGGCTACGTCCAGGA

GCGCACCATCTTCTTCAAGGACGACGGCAACTACAAGACCCGCGCCGAGG

TGAAGTTCGAGGGCGACACCCTGGTGAACCGCATCGAGCTGAAGGGCATC

GACTTCAAGGAGGACGGCAACATCCTGGGGCACAAGCTGGAGTACAACTA

CAACAACCACAACGTCTATATCATGGCCGACAAGCAGAAGAACGGCATCA

AGGTGAACTTCAAGATCCGCCACAACATCGAGGACGGCAGCGTGCAGCTC

GCCGACCACTACTAGCAGAACACCCCCATCGGCGACGGCCCCGTGCTGCT

GCCCGACAACCACTACCTGAGCACCCAGTCCGCCCTGAGCAAAGACCCCA

ACGAGAAGCGCGGTCACATGGTCCTGCTGGAGTTCGTGACCGCCGCCGGG

ATCACTCTCGGCATGGACGAGCTGTACAAGTAAAGCGGCCGCGACTCTAG

ATCATAATCAGC

>E31

GGATCCACCGGTCGCCACCATGGTGAGCAAGGGCGAGGAGCTGTTCACTG

GGGTGGTGCCCATCCTGGTCGACGGCGACATAAACGGCCACAAGTTCAGC

GTGTCCGGCGAGGGCGGGGGCGATGCCACCTACGGCAAGCTGACCCTGAA

GTTCATCTGCACCACCGGCAAGCTGCCCGTGCCCTGGCCCACCCTCGTGA

CCACCCTGACCTACGGCGTGAAGTGCTTCAGCCGCTACCCCGACCACATG

AAGCAGCACGACTTCTTCAAGTCCGCCATGCCCGAAGGCTACGTCCAGGA

GCGCACCATCTTCTTCAAGGACGACGGCAACTGCAAGACCCGCGCCGAGG

TGAAGTTCGAGGGCGACACCCTGGTGAACCGCATCGAGCTGAAGGGCATC

GACTTCAAGGAGGACGGCAACATCCTGGGGCACAAGCTGGAGTACAACTA

CAACAGCCACAACGTCTATATCATGGCCGACAAGCAGAAGAACGGCATCA

AGGTGAACTTCAAGATCCGCCACAACATCGAGGACGGCAGCGTGCAGCTC

GCCGACCACTACCAGCAGAACACCCCCATCGGCGACGGCCCCGTGCTGCT

GCCCGACAACCACTACCTGAGCACCCAGTCCGCCCTGAGCAAAGACCCCA

ACGAGAAGCGCGGTCACATGGTCCTGCTGGAGTTCGTGACCGCCGCCGGG

ATCAGTCTCGGCATGGACGAACTGTACAAGTAAAGCGGCCGCGACTCTAG

ATCATAATCAGC

>E32

GGATCCACAGGTCGCCACCATGGTGAGCAAGGGCGAGGAGCTGTTCACTG

GGGTGGTGCCCATCCTGGTCGACGGCGACATAAACGGCCACAAGTTCAGC

GTGTCCGGCGAGGGCGGGGGCGATGCCACCTACGGCAAGCTGACCCTGAA

GTTCATCTGCACCACCGGCAAGCTGCCCGTGCCCTGGCCCACCCTCGTGA

CCACCCTGACCTACGGCGTGCAGTGCTTCAGCCGCTACCCCGACCACATG

AAGCAGCACGACTTCTTCAAGTCCGCCATGCCCGAAGGCTACGTCCAGGA

GCGCACCATCTCCTTCAAGGACGACGGCAACTACAAGACCCGCGCCGAGG

TGAAGTTCGAGGGCGACACCCTGGTGAACCGCATCGAGCTGAAGGGCATC

GACTTCAAGGAGGACGGCAACATCCTGGGGCACAAGCTGGAGTACAACTA

CAACAGCCACAACGTCTATATCATGGCCGACAAGCAGAAGAACGGCATCA

AGGTGAACTTCAAGATCCGCCACAACATCGAGGACGGCAGCGTGCAGCTC

GCCGACCACTACCAGCAGAACACCCCCATCGGCGACGGCCCCGTGCTGCT

GCCCGACAACCACTACCTGAGCACCCAGTCCGCCCTGGGCAAAGACCCCA

ACGAGAAGCGCGGTCACATGGTCCTGCTGGAGTTCGTGACCGCCGCCGGG

ATCACTCTCGGCATGGACGAGCTGTACAAGTAAAGCGGCCGCGACTCTAG

ATCATAATCAGC

>E34

GGATCCACCGGTCGCCACCATGGTGAGCAAGGGCGAGGAGCTGTTCACTG

GGGTGGTGCCCATCCTGGTCGACGGCGACATAAACGGCCACAAGTTCAGC

GTGTCCGGCGAGGGCGGGGGCGATGCCACCTACGGCAAACTGACCCTGAA

GTTCATCTGCACCACCGGCAAGCTGCCCGTGCCCTGGCCCACCCTCGTGA

CCACCCTGACCTACGGCGTGCAGTGCTTCAGCCGCTACCCCGACCACATG

AAGCAGCACGACTTCTTCAAGTCCGCCATGCCCGAAGGCTACGTCCAGGA

GCGCACCATCTTCTTCAAGGACGACGGCAACTACAAGACCCGCGCCGAGG

TGAAGTTCGAGGGCGACACCCTGGTGAACCGCATCGAGCTGAAGGGCATC

GACTTCAAGGAGGACGGCAACATCCTGGGGCACAAGCTGGAGTACAACTA

CAACAGCCACAACGTCTATATCATGGCCGACAAGCAGAAGAACGACATCA

AGGTGAACTTCAAGATCCGCCACAACATCGAGGACGGCAGCGTGCAGCTC

GCCGACCACTACCAGCAGAACACCCCCATCGGCGACGGCCCCGTGCTGCT

GCCCGACAACCACTACCTGAGCACCCAGTCCGCCCTGAGCAAAGACCCCA

ACGAGAAGCGCGGTCACATGGTCCTGCTGGAGTTCGTGACCGCCGCCGGG

ATCACTCTCGGCATGGACGAGCTGTACAAGTAAAGCGGCCGCGACCCTAG

ATCATAATCAGC

>E35

GGATCCACCGGTCGCCACCATGGTGAGCAAGGGCGAGGAGCTGTTCACTG

GGGTGGTGCCCATCCTGGTCGACGGCGACATAAACGGCCACAAGTTCAGC

GTGTCCGGCGAGGGCGGGGGCGATGCCACCTACGGCAAGCTGACCCTGAA

GTTCATCTGCACCACCGGCAAGCTGCCCGTGCCCTGGCCCACCCTCGTGA

CAACCCTGACCTACGGCGTGCAGTGCTTCAGCCGCTACCCCGACCACATG

CAGCAGCACGACTTCTTCAAGTCCGCCATGCCCGAAGGCTACGTCCAGGA

GCGCACCATCTTCTTCAAGGACGACGGCAACTACAAGACCCGCGCCGAGG

TGAAGTTCGAGGGCGACACCCTGGTGAACCGCATCGAGCTGAAGGGCATC

GACTTCAAGGAGGACGGCAACATCCTGGGGCACAAGCTGGAGTACAACTA

CAACAGCCACAACGTCTATATCATGGCCGACAAGCAGAAGAACGGCATCA

AGGTGAACTTCAAGATCCGCCACAACATCGAGGACGGCAGCGTGCAGCTC

GCCGACCACTACCAGCAGAACACCCCCATCGGCGACGGCCCCGTGCTGCT

GCCCGACAACCACTACCTGAGGACCCAGTCCGCCCTGAGCAAAGACCCCA

ACGAGAAGCGCGGTCACATGGTCCTGCTGGAGTTCGTGACCGCCGCCGGG

ATCACTCTCGGCATGGACGAGCTGTACAAGTAAAGCGGCCGCGACTCTAG

ATCATAATCAGC

>E36

GGATCCACCGGTCGCCACCATGGTGAGCAAGGGCGAGGAGCTGTTCACTG

GGGTGGTGCCCATCCTGGTCGACGGCGACATAAACGGCCACAAGTTCAGC

GTGTCTGGCGAGGGCGGGGGCGATGCCACCTACGGCAAGCTGACCCTGAA

GTTCATCTGCACCACCGGCAAGCTGCCCGTGCCATGGCCCACCCTCGTGA

CCACCCTGACCTACGGCGTGCAGTGCTTCAGCCGCTACCCCGACCACATG

AAGCAGCACGACTTCTTCAAGTCCGCCATGCCCGAAGGCTACGTCCAGGA

GCGCACCATCTTCTTCAAGGACGACGGCAACTACAAGACCCGCGCCGAGG

TGAAGTTCGAGGGCGACACCCTGGTGAACCGCATCGAGCTAAAGGGCATC

GACTTCAAGGAGGACGGCAACATCCTGGGGCACAAGCTGGAGTACAACTA

CAACAGCCACAACGTCTATATCATGGCCGACAAGCAGAAGAACGGCATCA

AGGTGAACTTCAAGATCCGCCACAACATCGAGGACGGCAGCGTGCAGCTC

GCCGACCACTACCAGCAGAACACCCCCATCGGCGACGGCCCCGTGCTGCT

GTCCGACAACCACTACCTGAGCACCCAGTCCGCCCTGAGCAAAGACCCCA

ACGAGAAGCGCGGTCACATGGTCCTGCTGGAGTTCGTGACCGCCGCCGGG

ATCACACTCGGCATGGACGAGCTGTACAAGTAAAGCGGCCGCGACTCTAG

ATCATAATCAGC

>E37

GGATCCACCGGTCGCCACCATGGTGAGCAAGGGCGAGGAGCTGTTCACTG

GGGTGGTGCCCATCCTGGTCGACGGCGACATAAACGGCCACAAGTTCAGC

GTGTCCGGCGAGGGCGGGGGCGATGCCACCTACGGCAAGCTGACCCTGAA

GTTCATCTGCACCACCGGCAAGCTGCCCGTGCCCTGGCCCACCCTCGTGA

CCACCCTGACCTACGGCGTGCAGTGCTTCAGCCGCTACCCCGACCACATG

AAGCAGCACGACTTCTTCAAGTCCGCCATGCCCGAAGGCTACGTCCAGGA

GCGCACCATCATCTTCAAGGACGACGGCAACTACAAGACCCGCGCCGAGG

TGAAGTTCGAGGGCGACACCCTGATGAACCGCATCGAGCTGAAGGTCATC

GACTTCAAGGAGGACGGCAACATCCTGGGGCACAAGCTGGAGTACTACTA

CAACAGCCACAACGTCTATCTCATGGCCGACAAGCAGAAGAACGGCATCA

AGGTGAACTTCAAGATCCGCCACAACATCGAGGACGGCAGCGTGCAGCTC

GCCGATCACTACCAGCAGAACACCCCCATCGGCGACGGCCCCGTGCTGCT

GCCCGACAACCACTACCTGAGCACCCAGTCCGCCCTGAGCAAAGACCCCA

ACGAGAAGCGCGGTCACATGGTCCTGCTGGAGTTCGTGACCGTCGCCGGG

ATCACTCTCGGCATGGACGAGCTGTACAAGTAAAGCGGCCGCGACTCTAG

ATCATAATCAGC

>E38

GGATCCACCGGTCGCAACCATGGTGAGCAAGGGCGAGGAGCTGTTCACTG

GGGTGGTGCCCATCCTGGTCGACGGCGACATAAACGGCCACAAGTTCAGC

GTGTCCGGCGAGGGCGGGGGCGATGCCACCTACGGCAAGCTGACCCTGAA

GTTCATCTGCACCACCGGCAAGCTGCCCGTGCCCTGACCCACCCTCGTGA

CCACCCTGACCTACGGCGTGCAGTGCTTCAGCCGCTACCCCGACCACATG

AAGCAGCACGACTCCTTCAAGTCCGCCATGCCCGAAGGCTACGTCCAGGA

GCGCACCATCTTCTTCAAGGACGACGGCAACTACAAGACCCGCGCCGAGG

TGAAGTTCGAGGGCGACACCCTGGTGAACCGCATCGAGCTGAAGGGCATC

GACTTCAAGGAGGACGGCAACATCCTGGGGCACAAGCTGGAGTACAACTA

CAACAGCCACAACGTCTATATCATGGCCGACAAGCAGAAGAACGGCATCA

AGGTGAACTTCAAGATCCGCCACAACATCGAGGACGGCAGCGTGCAGCTC

GCCGACCACTACCAGCAGAACACCCCCATCGGCGACGGCCCCGTGCTGCT

GCCCGACAACCACTACCTGAGCATCCAGTCCGCCCTGAGCAAAGACCCCA

ACGAGAAGCGCGGTCACATGGTCCTGCTGGAGTTCGTGACCGCCGCCGGG

ATCACTCTCGGCATGGACGAGCTGTACAAGTAAAGCGGCCGCGACTCTAG

ATCATAATCAGC

>E39

GGATCCACCGGTCGCCACCATGGCGAGCAAGGGCGAGGAGCTGTTCACTG

GGGTGGTGCCCATCCTGGTCGACGGCGACATAAACGGCCACAAGTTCAGC

GTGTCCGGCGAGGGCGGGGGCGATGCCACCTACGGCAAGCTGACCCTGAA

GTTCATCTGCACCACCGGCAAGCTGCCCGTGCCCTGGCCCACCCTCGTGA

CCACCCTGACCTACGGCGTGCAGTGCTTCAGCCGCTACCCCGACCACATG

AAGCAGCACGACTTCTTCAAGTCCGCCATGCCCGAAGGCTACGTCCAGGA

GCGCACCATCTTCTTCAAGGACGACGGCAACTACAAGACCCGCGCCGAGG

TGAAGTTCGAGGGCGACACCCTGGTGAACCGCATCGAGCTGAAGGGCATC

GACTTCAAGGAGGACGGCAACATCCTGGGGCACAAGCTGGAGTACAACTA

CAACAGCCACAACGTCTATATCATGGCCGACAAGCAGAAGAACGGCATCA

AGGTGAACTTCAAGATCCGCCACAACATCGAGGACGGCAGCGTGCAGCTC

GCCGACCACTACCAGCAGAACACCCCCATCGGCGACGGCCCCGTGCTGCT

GCCCGACAACCACTACCTGAGCACCCAGTCCGCCCTGAGCAAAGACCCCA

ACGAGAAGCGCGGTCACATGGTCCTGCTGGAGTTCGTGACCGCTGCCGGG

ATCACTCTCGGCATGGACGAGCTGTACAAGTAAAGCGGCCGCGACTCTAG

ATCATAATCAGC

>E40

GGATCCACCGGTCGCCACCATGGTGAGCAAGGGCGAGGAGCTGTTCACTG

GGGTGGTGCCCATCCTGGTCGACGGCGACATAAACGGCCACAAGTTCAGC

GTGTCCGGCGAGGGCGGGGGCGATGCCACCTACGGCAAGCTGACCCTGAA

GTTCATCTGCACCACCGGCAAGCTGCCCGTGCCCTGGCCCACCCTCGTGA

CCACCCTGACCTACGGAGTGCAGTGCTTCAGCCGCTACCCCGACCACATG

AAGCAGCACGACTTCTTCAAGTCCGCCATGCCCGAAGGCTACGTCCAGGA

GCGCACCATCTTCTTCAAGGACGACGGCAACTACAAGACCCGCGCCGAGG

TGAAGTTCGAGGGCGACACCCTGGTGAACCGCATCGAGCTGAAGGGCATC

GACTTCAAGGAGGACGGCAACATCCTGGGGCACAAGCTGGAGTACAACTA

CAACAGCCACAACGTCTATATCATGGCCGACAAGCAGAAGAACGGCATCA

AGGTGAACTTCAAGATCCGCCACAACATCGAGGACGGCAGCGTGCAGCTC

GCCGACCACTACCAGCAGAACACCCCCATCGGCGACGGCCCCGTGCTGCT

GCCCGACAACCACTACCTGAGCACCCAGTCCGCCCTGAGCAAAGACCCCA

ACGAGAAGCGCGGTCACATGGTCCTGCTGGAGTTCGTGACCGCCGCCGGG

ATCACTCTCGGCATGGACGAGCTGTACAAGTAAAGCGGCCGCGACTCTAG

ATCATAATCAGC

>E41

GGATCCACCGGTCGCCACCATGGTGAGCAAGGGCGAGGAGCTGTTCACTG

GGGTGGTGCCCATCCTGGTCGACGGCGACATAAACGGCCACAAGTTCAGC

GTGTCCGGCGAGGGCGGGGGCGATGCCACCTACGGCAAGCTGACCCTGAA

GTTCATCTGCACCACCGGCAAGCTGCCCGTGCCCTGGCCCACCCTCGTGA

CCACCCTGACCTACGGCGTGCAGTGCTTCAGCCGCTACCCCGACCACATG

AAGCAGCACGACTTCTTCAAGTCCGCCATGCCCGAAGGCTACGTCCAGGA

GCGCACCATCTTCTTCAAGGACGACGGCAACTACAAGACCCGCGCCGAGG

TGAAGTTCGAGGGCGACACCCTGGTGAACCGCATCGAGCTGAAGGGCATC

GACTTCAAGGAGGACGGCAACATCCTGGGGCACAAGCTGGAGTACAACTA

CAACAGCCACAACGTCTATTTCATGGCCGACAAGCAGAAGAACGGCATCA

AGGTGAACTTCAAGATCCGCCACAACATCGAGGACGGCAGCGTGCAGCTC

GCCGACCACTACCAGCAGAACACCCCCATCGGCGACGGCCCCGTGCTGCT

GCCCGACAACCACTACCTGAGCACCCAGTCCGCCCTGAGCAAAGACCCCA

ACGAGAAGCGCGGTCACATGGTCCTGCTGGAGTTCGTGACCGCCGCCGGG

ATCACTCTCGGCATGGACGAGTTGTTCAAGTAAAGCGGCCGCGACTCTAG

ATCATAATCAGC

>E42

GGATCCACCGGTCGCCACCATGGTGAGCAAGGGCGAGGAGCTGTTCACTG

GGGTGGTGCCCATCCTGGTCGACGGCGACATAAACGGCCACAAGTTCAGC

GTGTCCGGCGAGGGCGGGGGCGATGCCACCTACGGCAAGCTGACCCTGAA

GTTCATCTGCACCACCGGCAAGCTGCCCGTGCCCTGGCCCACCCTCGTGA

CCACCCTGACCTACGGCGTGCAGTGCTTCAGCCGCTACCCCGACCACATG

AAGCAGCACGACTTCTCCAAGTCCGCCATGCCCGAAGGCTACGTCCAGGA

GCGCACCATCTTCTTCAAGGACGACGGCAACTACAAGACCCGCGCCGAGG

TGAAGTTCGAGGGCGACACCCTGGTGAACCGCATCGAGCTGAAGGGCATC

GACTTCAAGGAGGACGGCAACATCCTGGGGCACAAGCTGGAGTACAACTA

CAACAGCCACAACGTCTATATCATGGCCGACAAGCAGAAGAACGGCATCA

AGGTGAACTTCAAGATCCGCCACAACATAGAGGACGGCAGCGTGCAGCTC

GCCGACCACTACCAGCAGAACACTCCCATCGGCGACGGCCCCGTGCTGCT

GCCCGACAACCACTACCTGAGCACCCAGTCCGCCCTGAGCAAAGACCCCA

ACGAGAAGCGCGGTCACATGGTCCTGCTGGAGTTCGTGACCGCCGCCGGG

ATCACTCTCGGCATGGACGAGCTGTACAAGTAAAGCGGCCGCGACTCTAG

ATCATAATCAGC

>E44

GGATCCACCGGTCGCCACCATGGTGAGCAAGGGCGAGGAGCTGTTCACTG

GGGTGGTGCCCATCCTGGTCGACGGCGACATAAACGGCAACAAGTTCAGC

GTGTCCGGCGAGGGCGGGGGCGATGCCACCTACGGCAAGCTGACCCTGAA

GTTCATCTGCACCACCGGCAAGCTGCCCGTGCCCTGGCCCACCCTCGTGA

CCACCCTGACCTACGGCGTGCAGTGCTTCAGCCGCTACCCCTACCACATG

AAGCAGCACGACTTCTTCAAGTCCGCCATGCCCGAAGGCTACGTCCAGGA

GCGCACCATCTACTTCAAGGACGACGGCAACTACAAGACCCGCGCCGAGG

TGAAGTTCGAGGGCGACACCCTGGTGAACCGCATCGAGCTGAAGGGCATC

GACTTCAAGGAGGACGGCAATATCCTGGGGCACAAGCTGGAGTACAACTA

CAACAGCCACAACGTCTATATCATGGCCGACAAGCAGAAGAACGGCATCA

AGGTGAGCTTCAAGATCCGCCACAACATCGAGGACGGCAGCGTGCAGCTC

GCCGACCACTACCAGCAGAACACCCCCATCGGCGACGGCCCCGTGCTGCT

GCCCGACAACCACTACCTGAGCACCCAGTCCGCCCTGAGCAAAGACCCCA

ACGAGAAGCGCGGTCACATGGTCCTGCTGGAGTTCGTGACCGCCGCCGGG

ATCACTCTCGGCATGGACGAGCTGTACAAGTAAAGCGGCCGCGACTCTAG

ATCATAATCAGC

>HEX_oligo_for_methyl_test

TTCAAGGATCTTACCGCTGTTGAGATCCAGTTCGATGTAACTTACTCCAC

TCGGGCGC

**8mer_loop_library**

>WLRC-1

GCGCCCGAGTGGTGCCGGCTGTTACATCGAACTGGATCTCAACAGCGGTAAGATCCTTGAA

>WLRC-2

GCGCCCGAGTGGATTGGGCGGTTACATCGAACTGGATCTCAACAGCGGTAAGATCCTTGAA

>WLRC-3

GCGCCCGAGTGGTGCCCCTCGTTACATCGAACTGGATCTCAACAGCGGTAAGATCCTTGAA

>WLRC-4

GCGCCCGAGTGGTGCCAGGTGTTACATCGAACTGGATCTCAACAGCGGTAAGATCCTTGAA

>WLRC-5

GCGCCCGAGTGGAGTCAATAGTTACATCGAACTGGATCTCAACAGCGGTAAGATCCTTGAA

>WLRC-6

GCGCCCGAGTGGGGCATGCGGTTACATCGAACTGGATCTCAACAGCGGTAAGATCCTTGAA

>WLRC-7

GCGCCCGAGTGGTCTTGCAAGTTACATCGAACTGGATCTCAACAGCGGTAAGATCCTTGAA

>WLRC-9

GCGCCCGAGTGGAACTTAGCGTTACATCGAACTGGATCTCAACAGCGGTAAGATCCTTGAA

>WLRC-10

GCGCCCGAGTGGCTAACACTGTTACATCGAACTGGATCTCAACAGCGGTAAGATCCTTGAA

**5mer_loop_oligos**

**In bold-actual, selection module sequences**

**>Olig_No_Loop**

**AGCTTTCAAGGATCTTACCGCTGTTGAGATCCAGTTCGATGTAACCCACTCGG**

>Olig_with_Loop

AGCTTTCAAGGATCTTACCGCTGTTGAGATCCAGTTCGATGTAACTTACTCCACTCGG

>Olig_No_Loop_RevComp

GCGCCCGAGTGGGTTACATCGAACTGGATCTCAACAGCGGTAAGATCCTTGAA

**>Olig_With_Loop_RevComp**

**GCGCCCGAGTGGAGTAAGTTACATCGAACTGGATCTCAACAGCGGTAAGATCCTTGAA**

>beta-Lactamase

TTACCAATGCTTAATCAGTGAGGCACCTATCTCAGCGATCTGTCTATTTC

GTTCATCCATAGTTGCCTGACTCCCCGTCGTGTAGATAACTACGATACGG

GAGGGCTTACCATCTGGCCCCAGTGCTGCAATGATACCGCGAGACCCACG

CTCACCGGCTCCAGATTTATCAGCAATAAACCAGCCAGCCGGAAGGGCCG

AGCGCAGAAGTGGTCCTGCAACTTTATCCGCCTCCATCCAGTCTATTAAT

TGTTGCCGGGAAGCTAGAGTAAGTAGTTCGCCAGTTAATAGTTTGCGCAA

CGTTGTTGCCATTGCTACAGGCATCGTGGTGTCACGCTCGTCGTTTGGTA

TGGCTTCATTCAGCTCCGGTTCCCAACGATCAAGGCGAGTTACATGATCC

CCCATGTTGTGCAAAAAAGCGGTTAGCTCCTTCGGTCCTCCGATCGTTGT

CAGAAGTAAGTTGGCCGCAGTGTTATCACTCATGGTTATGGCAGCACTGC

ATAATTCTCTTACTGTCATGCCATCCGTAAGATGCTTTTCTGTGACTGGT

GAGTACTCAACCAAGTCATTCTGAGAATAGTGTATGCGGCGACCGAGTTG

CTCTTGCCCGGCGTCAATACGGGATAATACCGCGCCACATAGCAGAACTT

TAAAAGTGCTCATCATTGGAAAACGTTCTTCGGGGCGAAAGCTTTCAAGG

ATCTTACCGCTGTTGAGATCCAGTTCGATGTAACCCACTCGGGCGCCCAA

CTGATCTTCAGCATCTTTTACTTTCACCAGCGTTTCTGGGTGAGCAAAAA

CAGGAAGGCAAAATGCCGCAAAAAAGGGAATAAGGGCGACACGGAAATGT

TGAATACTCAT

>Kanamycin_resistance_gene

TACATTCAAATATGTATCCGCTCATGAATTAATTCTTAGAAAAACTCATC

GAGCATCAAATGAAACTGCAATTTATTCATATCAGGATTATCAATACCAT

ATTTTTGAAAAAGCCGTTTCTGTAATGAAGGAGAAAACTCACCGAGGCAG

TTCCATAGGATGGCAAGATCCTGGTATCGGTCTGCGATTCCGACTCGTCC

AACATCAATACAACCTATTAATTTCCCCTCGTCAAAAATAAGGTTATCAA

GTGAGAAATCACCATGAGTGACGACTGAATCCGGTGAGAATGGCAAAAGT

TTATGCATTTCTTTCCAGACTTGTTCAACAGGCCAGCCATTACGCTCGTC

ATCAAAATCACTCGCATCAACCAAACCGTTATTCATTCGTGATTGCGCCT

GAGCGAGACGAAATACGCGGTCGCTGTTAAAAGGACAATTACAAACAGGA

ATCGAATGCAACCGGCGCAGGAACACTGCCAGCGCATCAACAATATTTTC

ACCTGAATCAGGATATTCTTCTAATACCTGGAATGCTGTTTTCCCTGGGA

TCGCAGTGGTGAGTAACCATGCATCATCAGGAGTACGGATAAAATGCTTG

ATGGTCGGAAGAGGCATAAATTCCGTCAGCCAGTTTAGTCTGACCATCTC

ATCTGTAACATCATTGGCAACGCTACCTTTGCCATGTTTCAGAAACAACT

CTGGCGCATCGGGCTTCCCATACAATCGATAGATTGTCGCACCTGATTGC

CCGACATTATCGCGAGCCCATTTATACCCATATAAATCAGCATCCATGTT

GGAATTTAATCGCGGCCTAGAGCAAGACGTTTCCCGTTGAATATGGCTCA

TACTCTTCCTTTTTCAATATTATTGAAGCATTTATCAGGGTTATTGTCTC

ATGAGCGGATACATATTTGAATGTA
