# Supplementary material for: Programmable In Vivo Selection of Arbitrary DNA Sequences
Source: PLoS One. 2012 Nov 14;7(11):e47795. doi: 10.1371/journal.pone.0047795 (PMC3498277; doi:10.1371/journal.pone.0047795)
Supplement: Text S3 — In silico simulation of enrichment potential. (DOC) [file pone.0047795.s012.doc]

***In silico* simulation of enrichment potential**

Our synthetic device is designed to positively select for homo-duplex input modules and negatively select for hetero-duplex (mismatched) input modules. The enrichment generated by this re-annealing phase (See Figure 2) forms the basis upon which the bacterial MMR system distinguishes between the correct and incorrect input modules.

We simulated the process of re-annealing of the input module in order to evaluate our devices potential for enriching the homo-duplex fraction of a specific input module variant compared to other variants in the library. Largely, two parameters govern the enrichment factor for any given error free input module within a library of input modules: (1) its initial fraction compared to the pool of all other erroneous input modules and (2) the number of different erroneous input modules. Our simulation shows that under specific population regimes the re-annealing of input modules significantly enriches the homo-duplex fraction of a specific input module variant (Figure S1). The results of this simulation show that, within these population constraints, the re-annealed devices are compatible with and should theoretically support MMR mediated enrichment of a specific input module according to the scheme we describe (Figure 1).
