# Supplementary material for: Programmable In Vivo Selection of Arbitrary DNA Sequences
Source: PLoS One. 2012 Nov 14;7(11):e47795. doi: 10.1371/journal.pone.0047795 (PMC3498277; doi:10.1371/journal.pone.0047795)
Supplement: Text S4 — Device operation in wild type Vs. Dam deficient bacteria - In vivo Dam methylation may reprogram the device. (DOC) [file pone.0047795.s013.doc]

**Device operation in wild type Vs. Dam deficient bacteria - *In vivo* Dam methylation may reprogram the device**

We suspected that the natural Dam methylase of wild type *E.coli* may fully methylate the two hemimethylated GATC sites on the Selection module prior to MMR processing , thereby eliminating its essential role in establishing strand selection and genotype switching in our device. Therefore, we compared the operation of the device upon transformation to wild type and Dam deficient *E.coli* (Figure S2), which has fully functional MMR machinery but lack Dam methylase. Our results show that, as we suspected, the enrichment potential of our device is indeed reduced in wild type bacteria compared to a Dam deficient GM48 strain. As a result, despite other potential drawbacks of the Dam deficient GM48 strain, we used it in all consequent experiments (Figure S2).

We wanted to compare Dam deficient and WT *E.coli* for their compatibility with the requirements of our device in vivo. The first strain is a standard cloning strain (E.Cloni) and the second one is Dam deficient GM48.

38 GFP variants with the same molarity were united with the reference GFP to reach a final 1:1 ratio (The ratio between the reference and each GFP variant is 38:1). Cloning of selection sites was done using selection sites: A (negative control) and C (activated device, Table S2). Colonies which grew under positive ampicillin selection were processed by colony PCR and sequencing. We analyzed sequences by two categories: Error free clones, which possess the reference sequence and clones with errors, which possess any other sequence from the 38 GFP variants. We defined the enrichment factor of the system as the ratio between the clones with error free GFP to all other clones which contain erroneous GFP sequences. We expected to find an enrichment factor of 1 from our negative control and larger than 1 for the operative device.

Dam (DNA adenine methylase) methylates hemimethylated adenine at GATC sites after replication. On the other hand, Dam- bacteria are exposed to other drawbacks which are considered: lower growth rate, lower transformation efficiency, and higher mutation rate than normal strains. With these considerations and the comparison results (Figure S2), we chose to continue working with GM48 as the strain for our system.

**Supplementary references**

1. Schaaper RM (1993) Base selection, proofreading, and mismatch repair during DNA replication in Escherichia coli. J Biol Chem 268: 23762-23765.
