# Supplementary material for: Programmable In Vivo Selection of Arbitrary DNA Sequences
Source: PLoS One. 2012 Nov 14;7(11):e47795. doi: 10.1371/journal.pone.0047795 (PMC3498277; doi:10.1371/journal.pone.0047795)
Supplement: Text S5 — Methylation evaluation experiment - Selection module hemi-methylation is highly efficient. (DOC) [file pone.0047795.s014.doc]

**Methylation evaluation experiment - Selection module hemi-methylation is highly efficient**

The strand selection activity of the Selection module depends on its GATC sites being efficiently hemi-methylated, since the bacterial MMR system relies on hemi-methylated GATC sites for strand selection. Therefore, we assayed the extent of the devices hemi-methylation at the Dam sites following chemical and enzymatic methylation with an enzymatic assay, which showed that methylation of the Dam sites is more efficient using chemical methylation (Figure S3).

The two hemi-methylated GATC sites of the Selection module were generated by chemically methylating oligos at the appropriate adenosine residues (the two Dam sites) and annealing them to their complementary un-methylated strand to form a hemimethylated Selection site. Chemical methylation was evaluated using an enzymatic assay.

We have designed an assay to test the methylation efficiency of in the Selection site. This assay uses MboI is a restriction enzyme which digests dam sites (GATC) but is blocked by methylated and hemimethylated dam sites.

We showed that the efficiency of the methylation in the modified oligonucleotide is almost 100%. We also checked the creation of methylation by in vitro methylation by dam Methyltransferase (NEB) but the results of the digestion showed that the efficiency of the in vitro methylation is not as good as the chemical methylation.
