# Supplementary material for: Programmable In Vivo Selection of Arbitrary DNA Sequences
Source: PLoS One. 2012 Nov 14;7(11):e47795. doi: 10.1371/journal.pone.0047795 (PMC3498277; doi:10.1371/journal.pone.0047795)
Supplement: Text S6 — Restriction control for the insertion of the Selection module. (DOC) [file pone.0047795.s015.doc]

**Restriction control for the insertion of the Selection module**

Insertion of the heteroduplex hemimethylated selection site requires proper digestion by two restriction enzymes. In order to make sure that both restriction enzymes properly digest the vector, a restriction enzymes control experiment was added, parallel to each cloning experiment (Figure S4).
