# Supplementary material for: Programmable In Vivo Selection of Arbitrary DNA Sequences
Source: PLoS One. 2012 Nov 14;7(11):e47795. doi: 10.1371/journal.pone.0047795 (PMC3498277; doi:10.1371/journal.pone.0047795)
Supplement: Text S7 — Selection module loop optimization - Integration of the Selection module loop into the device can be optimized. (DOC) [file pone.0047795.s016.doc]

**Selection module loop optimization - Integration of the Selection module loop into the device can be optimized**

We found that the size of the device library (i.e. number of devices with unique input modules) is limited by the cloning of the Selection module into the device. We therefore used an Amp resistance restoration colony counting assay to optimize this step and found two factors which increased its throughput: (1) placing the loop structure on the coding (as opposed to the non-coding) strand and (2) optimizing Selection module concentration during its ligation into the device (Figure S5, Table S3).

The experiments were performed using a plasmid population containing only one unique diagnostic site, no re-annealing was performed and no strand was methylated. Same volume of transformants was inoculated on both plates for each transformation. We have also verified the efficiency of supercoiled devices (Amp+ and Amp- which is the original pIVEC genotype) to perform their expected selection in an ampicillin containing medium (colonies formation & no colonies respectively). Experiments results have shown that loop structure A (according to Table S3) was more efficient in ampicillin resistance retrieval and was chosen to be the structure of our experiments (see sequences in Text S2).
