# Supplementary material for: Programmable In Vivo Selection of Arbitrary DNA Sequences
Source: PLoS One. 2012 Nov 14;7(11):e47795. doi: 10.1371/journal.pone.0047795 (PMC3498277; doi:10.1371/journal.pone.0047795)
Supplement: Text S8 — Invisibility of the selection module loop to the MMR system -The selection module loop is partially invisible to the MMR system. (DOC) [file pone.0047795.s017.doc]

**Invisibility of the selection module Loop to the MMR system - The selection module Loop is partially invisible to the MMR system**

For our synthetic device to function properly its heterozygous Selection module had to be invisible to the MMR system in the sense that its loop structure must not be detected as a mutation and repaired. Therefore, we designed the Selection module to have a 5bp loop structure which generally goes undetected by the MMR system . Additionally, we performed verification and optimization experiments in order to verify that the loop we designed onto the Selection module is indeed largely invisible to the bacterial MMR system.

In order to verify if this assumption works, we have designed and cloned a library of 9 different random sequences of 8-mer loop structures in which both strands have no function and confer no selective advantage upon transformation. All loops are flanked by the same sequence similar to the 5-mer loop structure region in the final design of our project. The idea behind the library test is to see whether bacteria cloned with looped structure similar to ours retain and propagate both types of DNA molecules (Figure S6). This would indicate that these structures are at least partially invisible to the MMR system. We cloned these structures by a standard T/A cloning procedure the cloning site of pGEM vector. In that region, both strands do not affect or confer any resistance if replicated. A preferred outcome will lead to colonies which confer two kinds of populations which imply that there is no strand selection or detection by MMR. We picked the best heteroduplex candidate which had 58% (28 of 48 colonies) success in forming colonies which possess two populations. Unfortunately, this heteroduplex showed no improvement in colony formation under ampicillin selection compared to the original 5nt heteroduplex.

**Supplementary references**

1. Parker BO, Marinus MG (1992) Repair of DNA heteroduplexes containing small heterologous sequences in Escherichia coli. Proc Natl Acad Sci U S A 89: 1730-1734.
