# Supplementary material for: Programmable In Vivo Selection of Arbitrary DNA Sequences
Source: PLoS One. 2012 Nov 14;7(11):e47795. doi: 10.1371/journal.pone.0047795 (PMC3498277; doi:10.1371/journal.pone.0047795)
Supplement: Text S9 — Generating the library of input modules using error-prone PCR. (DOC) [file pone.0047795.s018.doc]

**Generating the library of input modules using error-prone PCR**

In order to test the capacity of our synthetic device to enrich for a specific sequence within a library of many other sequences we generated a library of input molecules using error prone PCR (see Materials and Methods) of a GFP gene and clone sequenced 38 variants into the device as inputs (see sequences in Text S2). Each sequence was unique and contained 1-10 mutated bases compared to the reference sequence, with an average of 4.03 mutations per sequence (see mutation analysis of GFP variants compared with error-free GFP sequence reference in Table S1).
