# Supplementary material for: Programmable In Vivo Selection of Arbitrary DNA Sequences
Source: PLoS One. 2012 Nov 14;7(11):e47795. doi: 10.1371/journal.pone.0047795 (PMC3498277; doi:10.1371/journal.pone.0047795)
Supplement: Text S10 — Description of experimental procedures. (DOC) [file pone.0047795.s019.doc]

**Description of experimental procedures**

A DNA library to be screened and selected *in vivo* is cloned into the input module of the device and the entire device library is purified in one batch from bacteria. The device library is then linearized by double restriction at the Selection site. The linearized double stranded device is then subjected to heat renaturation, randomly annealing single strands from different devices in the library and exposing the mismatched bases between their input modules. As discussed earlier, our synthetic device selects against hetero-duplex input modules.

Re-annealing of the device under specific population regimes (depicted in Figure 3) converts almost all the population of devices (which are initially exclusively homo-duplex) to being hetero-duplex, with only a small fraction remaining homo-duplex molecules. Specifically, the outcome of this *in vitro* annealing step depends on two parameters, namely (1) the fraction of devices with the input sequence we want to enrich for out of all devices in the library and (2) the number of unique devices with input sequences which differ from the one we want to enrich for. Under certain input library population regimes (depicted in Figure 3) the re-annealing step creates two distinct populations (1) a homo-duplex DNA population which is highly enriched for a specific DNA sequence and is targeted for *in vivo* positive selection and (2) a second population of hetero-duplex DNA, which are targeted for negative *in-vivo* selection.

Specifically, the enrichment factor increases exponentially as the number of unique erroneous fragments increases and the fraction of the error free fragment decreases, within the boundaries we have tested. The same principle has been utilized for eliminating errors *in vitro* using Endonucleases.

The Selection module (See Figure 1b) is then ligated into the linearized, re-annealed device at the restriction site and the circular device is transformed into Dam- *E.coli*. We use Dam- bacteria since they lack GATC methylation activity, which eliminates the ability of the bacteria to modify the hemi-methylation pattern of the synthetic device.

Once cloned into a population of Dam- bacterial cells, the device undergoes a MutHLS-based positive or negative diagnosis based on the existence of mismatched DNA in the input module.

As discussed earlier, the DNA device is designed with two functional modules, namely the (1) 'input' and (2) 'Selection' segments. Upon introduction of the device to a bacterial cell the endogenous MutHLS machinery probes the input module and detects whether a mismatch exists in within it. Positive diagnosis resulting from mismatched input DNA results in further activation of the MMR which then searches for the closest hemi-methylated GATC site within a several Kb range and MutHLS mediated reprogramming of the device to an Amp- genotype, resulting in negative selection due to the presence of Ampicillin. Conversely, upon negative diagnosis of the input module (no mutation) the Selection module remains in its unmodified, heterozygous state resulting in positive selection under antibiotic selection results in cell survival.

The systems generic design allows it to be programmed with any DNA sequence as the input module and ultimately enriches its sequence within the bacterial population.

The system’s ability to use bacterial MMR for querying any input DNA sequence and selecting for error free ones enabled us to address the fundamental problem of DNA error correction in synthetic biology, previously addressed by us and others .

**Supplementary references**

1. Linshiz G, Ben Yehezkel T, Kaplan S, Gronau I, Ravid S, et al. (2008) Recursive construction of perfect DNA molecules from imperfect oligonucleotides. Molecular Systems Biology: -.

2. Carr P, Park J, Lee Y, Yu T, Zhang S, et al. (2004) Protein-mediated error correction for de novo DNA synthesis. Nucleic Acids Research: -.

3. Matzas M, Stähler PF, Kefer N, Siebelt N, Boisguérin V, et al. (2010) High-fidelity gene synthesis by retrieval of sequence-verified DNA identified using high-throughput pyrosequencing. Nat Biotechnol 28: 1291-1294.
